# Anionic Synthetic Polymers Prevent Bacteriophage Infection

**DOI:** 10.1021/jacs.3c01874

**Published:** 2023-04-17

**Authors:** Huba L. Marton, Peter Kilbride, Ashfaq Ahmad, Antonia P. Sagona, Matthew I. Gibson

**Affiliations:** † Department of Chemistry, University of Warwick, Gibbet Hill Road, Coventry CV4 7AL, U.K.; ‡ Division of Biomedical Sciences, Warwick Medical School, University of Warwick, Gibbet Hill Road, Coventry CV4 7AL, U.K.; § School of Life Sciences, University of Warwick, Coventry CV4 7A, U.K.; ∥ Asymptote, Cytiva, Chivers Way, Cambridge CB24 9BZ, U.K.

## Abstract

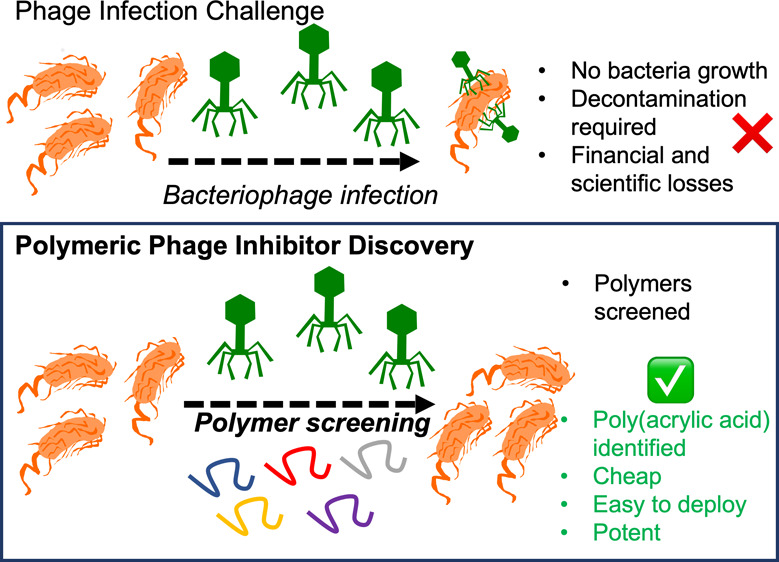

Bioprocessing and biotechnology exploit microorganisms (such as bacteria) for the production of chemicals, biologics, therapies, and food. A major unmet challenge is that bacteriophage (phage) contamination compromises products and necessitates shut-downs and extensive decontamination using nonspecific disinfectants. Here we demonstrate that poly(acrylic acid) prevents phage-induced killing of bacterial hosts, prevents phage replication, and that induction of recombinant protein expression is not affected by the presence of the polymer. Poly(acrylic acid) was more active than poly(methacrylic acid), and poly(styrenesulfonate) had no activity showing the importance of the carboxylic acids. Initial evidence supported a virustatic, not virucidal, mechanism of action. This simple, low-cost, mass-produced additive offers a practical, scalable, and easy to implement solution to reduce phage contamination.

It is now possible to edit biosynthetic pathways in bacteria to produce high-value chemicals and natural products.^1^ Bacteria are widely used in food production. For bacteria to be used in any application area, it is essential to exclude bacteriophage (phage–bacteria selective viruses), which are a common cause of infection that leads to financial and scientific losses. Bacteriophages are among the most abundant organisms on earth and are present wherever their hosts are.^2^ Phages have potential as alternatives to antibiotics^3−5^ for food safety^6^ and veterinary settings.^7^ Phages are also widely used in biotechnology for ligand selection^8−10^ and other areas.^8,11^

Despite their wide biotechnological use, phage contamination in bacterial cultures leads to a complete loss of the culture. This has significant cost implications for both academic and industrial laboratories that have invested in isolating and preparing these bacterial cultures. For example, in the food industry, it is not possible to remove all phage from raw materials, and this can lead to process collapse.^12−14^ Currently, good microbiology practice, aseptic conditions, and vigorous cleaning or autoclaving are the primary mitigation tools. These methods are not always successful, as phages are robust and can survive in almost every condition.^15^ One option is to engineer bacterial strains, which are intrinsically resistant to phage, using, for example, gene editing technology, but this is not trivial and might not be suitable for all hosts.^16^

Changing processes or re-engineering strains that have been optimized for a particular biorefinery challenge is not simple: a pragmatic solution would be an antiphage additive, in much the same way that antibiotics are routinely used in mammalian cell culture, to prevent bacterial infection.^17^ There are many studies on the use of phage in treatment^7,18,19^ and for ligand screening,^9,10,20^ but very few on tools to inhibit them. In contrast, mammalian viruses have been investigated for the discovery of viral inhibitors^21,22^ and for repurposing of existing inhibitors.^23^

Bacterial hosts have evolved alongside phages and hence have strategies to prevent/reduce phage infection, mostly relying on protein components, restriction-modification, and clustered regularly interspaced short palindromic repeats (CRISPR) defenses,^24−26^ which are not easy to repurpose as an antiphage additive. There are a small number of reports of molecules that can inhibit phage infection: those discovered in *Streptomyces*(27) and some aminoglycoside antibiotics.^28^ The latter are not desirable for large-scale biotechnological application due to antimicrobial resistance concerns. It has recently been demonstrated that sulfated polymers, which mimic heparin sulfate anchors on cell membranes, are broad-spectrum virucides against a range of human pathogenic viruses.^29,30^ Poly(carboxylic acid)s have been reported to inhibit human viruses.^31,32^ It is also well-established that polymers can be deployed as antibacterial agents, mimicking cationic host-defense peptides.^33−36^ A polymeric/biomaterials approach to address phage contamination may offer a scalable and practical solution. To the best of our knowledge, the only report of antiphage polymer is dextran, dextran sulfate, and polystyrene sulfate, which show some limited inhibition but have been neither widely explored nor compared to other polymers, and their mode of action is not studied.^37^

Here we report the novel discovery that poly(acrylic acid) (PAA) is a potent inhibitor of bacteriophage infection. A library of polymers was screened, showing this material to be uniquely active, even compared to other poly(carboxylic acid)s. The polymer prevents infection and is shown to not interfere with recombinant protein expression procedures in bacteria. This offers a scalable, practical, low-cost, and easy to deploy solution to the problem of phage contamination with no need to change user protocols.

To evaluate if a synthetic polymer could be discovered to inhibit bacteriophage infection, we prepared a panel of water-soluble polymers including neutral and anionic polymers. Cationic polymers were excluded, as they have antibacterial activity^38,39^ and are hence not compatible with the assays, which aim to allow bacteria to grow. The panels of polymers were prepared using RAFT (reversible addition–fragmentation chain transfer)^40^ polymerization to enable control over molecular weight and dispersity, Figure 1. All polymers were characterized by ^1^H NMR and (SEC) size exclusion chromatography, Table S1. Full experimental details of the polymer synthesis are in the Supporting Information. Polymers are referred to by their number-average degree of polymerization (DP), and molecular weight distributions are given in Figure 1B–F.

**Figure 1:**
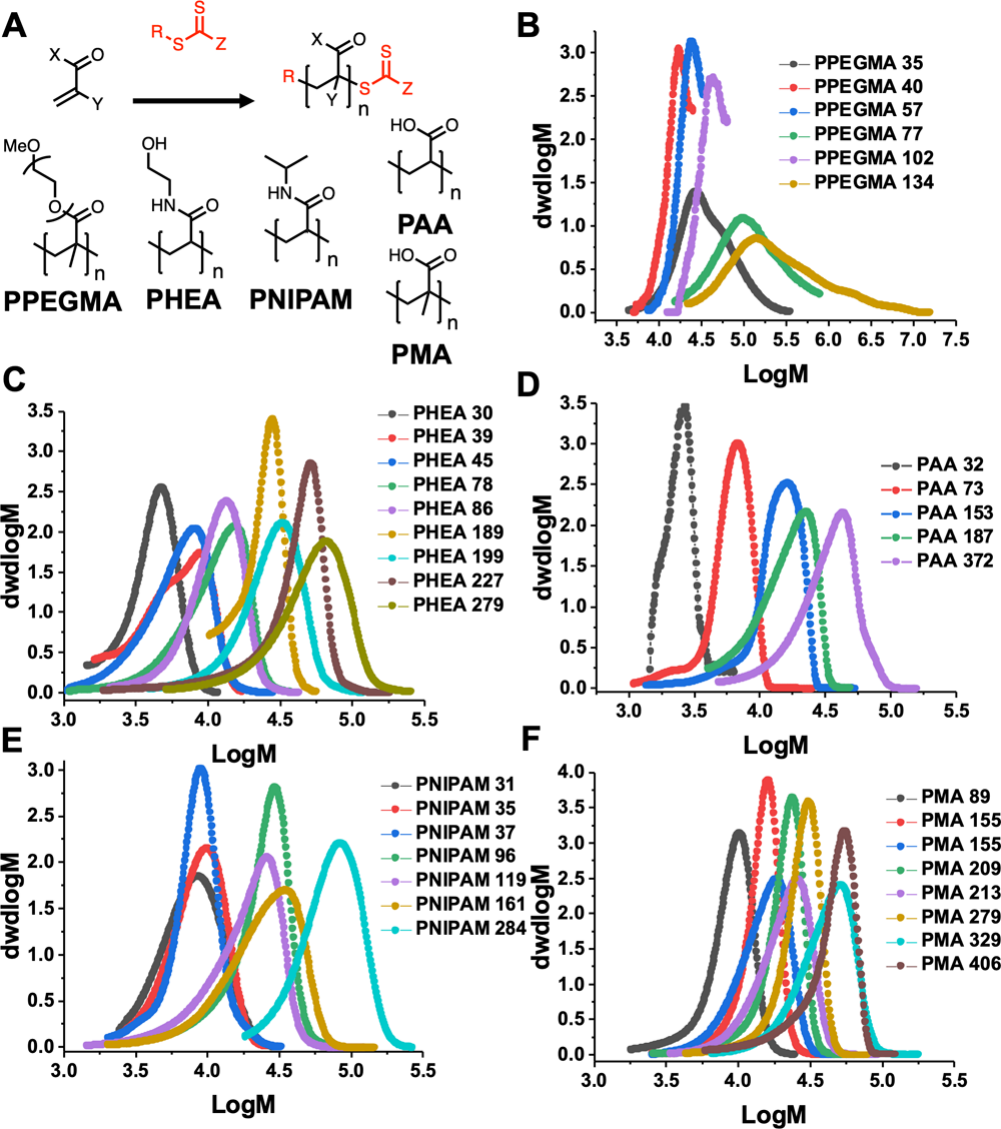
Polymers synthesized. (A) RAFT polymerization (full details in Supporting Information). Molecular weight distribution for (B) PPEGMA (poly(polyethylene glycol)methacrylate); (C) PHEA (poly(*N*-hydroxyethylacrylamide); (D) PAA (poly(acrylic acid); (E) PNIPAM (poly(*N*-isopropylacrylamide); (F) PMA (poly(methacrylic acid).

To screen for the unprecedented function of an antibacteriophage polymer, a high-throughput 96-well microplate-based assay was devised to maximize chemical space screening. In brief, polymers were serially diluted in the appropriate growth medium and added to the indicated bacteriophage. This was then added to a culture of *E. coli* (*Escherichia coli*) EV36 or *E. coli* K-12 (MG1655 cells) (depending on phage used) seeded at a density of 0.001 (1 × 10^6^ colony forming units (CFU·mL^–1^)) and incubated at 37 °C for 24 h. If the bacteria grow, there is an increase in OD600 (standard method for bacterial growth curves), Figure 2A. If the phages are viable, they will inhibit bacterial growth initially, before rebounding (as phages are not 100% effective at killing from a single dose). An example growth curve is shown in Figure 2B with poly(poly(ethylene glycol) methacrylate), PPEGMA, of different molecular weights, showing a decrease in OD600 after 4 h. This indicated that bacteriophages are viable and can kill the host, and hence PPEGMA is not having an impact on the phage. Controls of bacteria alone with all the polymers were conducted to ensure that there were no effects on bacterial growth. As might be expected, the vast majority of the polymers show no impact on the phage (i.e., bacteria are killed). However, there was one distinct exception: poly(acrylic acid), PAA. All molecular weights of PAA inhibited the action of the phage, and thus, the bacteria could grow, Figure 2C. This was surprising, considering that poly(methacrylic acid), PMA, was less effective unless higher concentrations were used, despite the minor structural difference of the backbone. This was a remarkable observation, as based on this screen this simple, low-cost, and widely used commodity polymer is capable of preventing phage infections from spreading in a bacterial culture.

**Figure 2:**
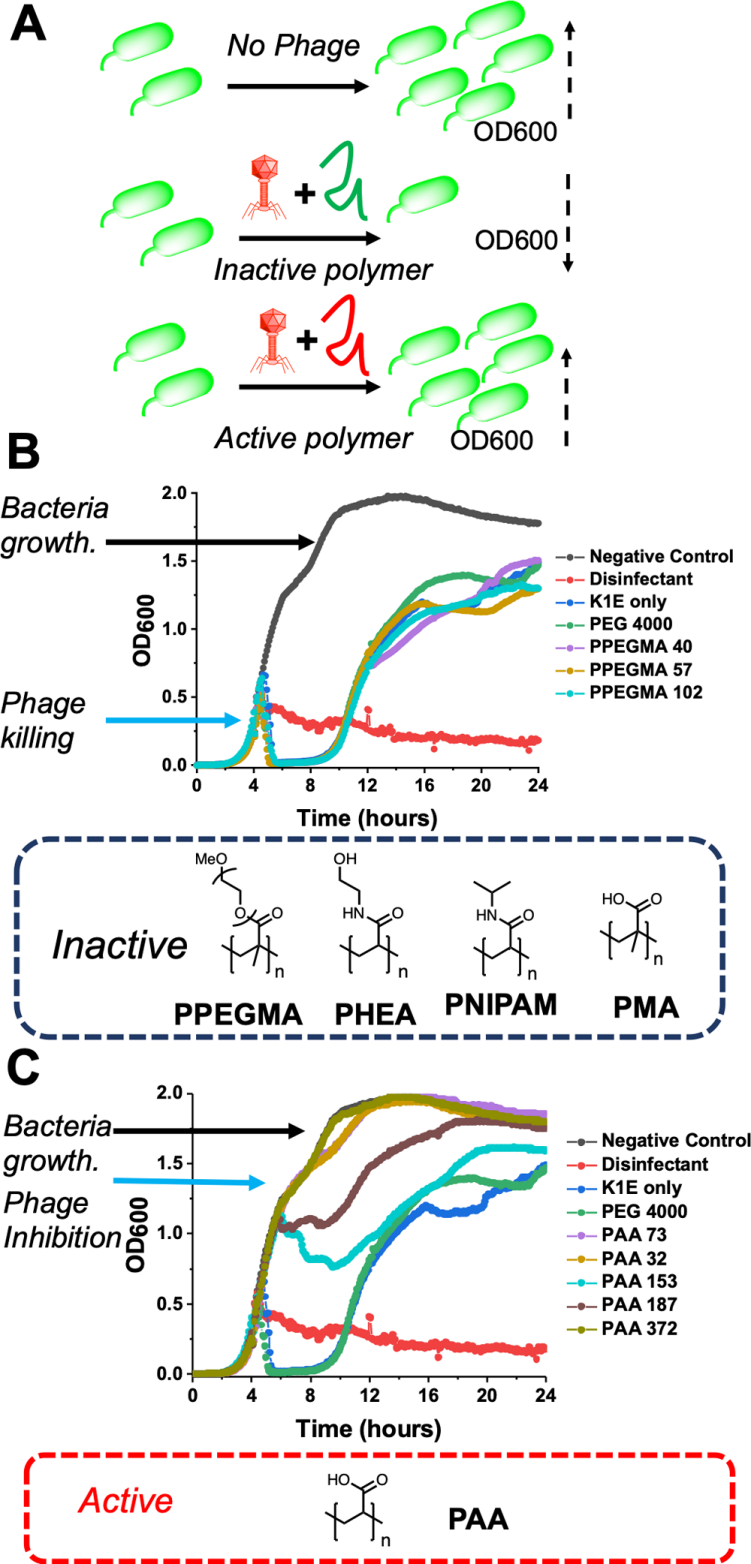
Screening for bacterial phage inhibition. (A) Concept of assay and polymer inhibition. (B) Representative growth curve with PPEGMA and inactive polymers from screening. (C) Representative growth curve with PAA, the only “hit” from the screen. *E. coli* EV36 was used as a bacterial host for the K1E bacteriophage. Complete screens are listed in Figures S4–S8.

PAA was further explored as a function of concentration from 10 mg·mL^–1^ and for all molecular weights. All of the phages were inhibited at 10 mg·mL^–1^, with no strong molecular weight dependence on this limit, although intermediate polymers around DP 100 appear to be slightly more active. At 2.5 mg·mL^–1^ the inhibitory activity decreased, and hence 10 mg·mL^–1^ (approximately 1 wt %) was deemed optimal. Table 1 shows the minimum inhibitory concentration (MIC) as a function of the phage type and polymer molecular weight.

**Table 1:** Minimum Inhibitory Concentration of PAAs from Solution-Phase Screening

	**Phage and MIC (mg·mL**^**–1**^**)**		
**Polymer**	**K1F**	**T4**	**T7**
PAA 32	10	>10	10
PAA 73	5	>10	5
PAA 153	5	>10	5
PAA 187	10	>10	10
PAA 372	10	>10	5

To further validate the above observations, we performed a plaque-counting assay. In this assay the phages are applied to *E. coli* on agar, allowing the total number of plaques formed to be counted, and it is more sensitive than the in situ growth curves. Figure 3A,B shows photographs of the agar after inoculation with the phage and PAA. Compared to the controls, there are clearly far fewer phage-associated plaques, with none visible in most cases. Figure 3C quantifies the plaques, confirming that, in the case of K1-GFP, K1E, K1–5, and T7, the PAA fully inhibited all bacteriophage growth. In the case of T4, visible plaques did form, but for PAA 73 and PAA 153, this was reduced from 10^9^ to 10^4^ PFUs, representing significant inhibition. Higher concentrations of PAA (20 mg·mL^–1^) fully inhibited this phage. One possible explanation for this difference is the actual phage loading in the experiment. One phage particle does not equate to 1 PFU, and the T4 phage might have more viral particles (and hence higher effective concentration). However, the T4 data did confirm that there is an optimal PAA molecular weight around DP of 100.

**Figure 3:**
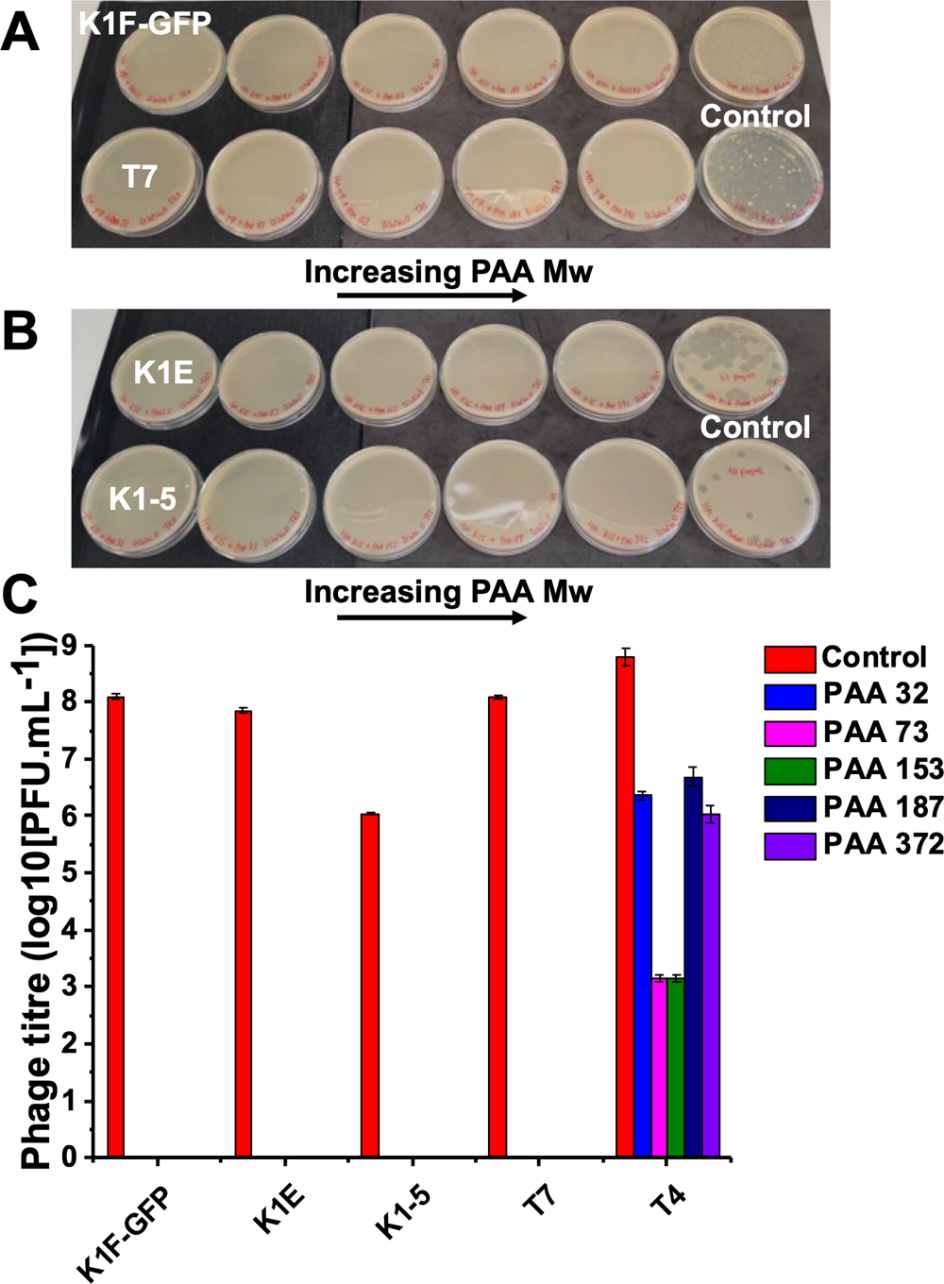
Poly(acrylic acid) bacteriophage plaque assay. (A, B) Photographs of plates showing reduction in plaques compared to nonpolymer control using indicated phage. (C) Quantification of plaque counting as a function of polymer molecular weight. *E. coli* EV36 was used as host for K1F-GFP, K1E, and K1–5 phages; *E. coli* MG1655 was used as host for T7 and T4 phages. 96 h incubation preplating. [polymer] = 10 mg·mL^–1^.

It is important to highlight that applying the polymers as a “therapeutic” (to bacteria already infected with phage) did not rescue bacterial growth (Figures S16 & S17). Hence this is a prophylactic strategy and suggests the polymer requires a certain amount of time to function and is acting on phage outside of the bacterial cells as part of its mode of action (discussed later).

Many mammalian viruses (but not known for phages) engage cell surface heparin sulfate, and polymeric sulfates have been found to be virucidal.^29,30,32^ PMA and PAA have been reported to inhibit human cell infection by mammalian viruses but have not been explored for bacteriophage inhibition.^31^ Dextran sulfate may partially inhibit phage infection,^37^ but the mechanism has not been explored. Here, poly(styrenesulfonate) was found to have no phage-inhibiting activity (Figure S18) in our assays, in contrast to mammalian viruses where polysulfonates are virucidal.^29^ Hence carboxylic acids, based on the basis of this first data set, seem to be the optimal anionic groups.

To probe the mechanism further, an experiment was devised to see if the polymers permanently (i.e., virucidal) or transiently (i.e., virustatic) inhibit the phage. K1F was incubated with 10 mg·mL^–1^ of PAA (concentration so no infection occurs) for 24 h. After this time, the phage/polymer solution was diluted so that the polymer was below its MIC (no inhibition in the standard experiments) before being added to the *E. coli* host. Upon dilution, the phages were able to eradicate the bacteria, equivalent to a control of untreated phage (at equal PFU/mL to account for dilution). This confirms a virusatic mechanism of action (Figure S14). PMA was also tested (at appropriate concentration to account for its lower activity), and a similar virustatic mechanism was observed (Figure S15). This is in contrast to anionic polymers, which inhibit zika virus, where increased hydrophobicity on the backbone increased activity, suggesting that prokaryotic and eukaryotic viruses require distinct polymers to inhibit them.^41^ The reduced activity of PMA may be linked to the fact that PAA/PMA do not have identical pH-dependent solution behavior (here pH 7.5 was used) but will need further investigation.^42^ Transmission electron microscopy (TEM) images of phages with PAA showed intact viruses (Figure S11) and some aggregates. However, dynamic light scattering did not show an increase in hydrodynamic diameter upon PAA/phage incubation (Figure S19), and hence we propose the polymer can reversibly bind the phage surface as a tentative mechanism of action.

For this technology to be broadly useful, it is important that the additives do not impact biotechnological protocols and, in particular, recombinant protein expression.^43^ Therefore, the impact of PAA on the expression of green fluorescent protein (GFP) was evaluated. PAA was added to a range of culture media for different *E. coli* BL21 (DE3) strains: wild-type untransformed lacking any plasmid; DE3 strain, which has been transformed with pWALDO plasmid encoding for GFP; and DE3 strain, which has been transformed with pT5T plasmid encoding for the human lectin DC-SIGN (but not GFP as a negative control). All strains were first tested against the previously used phage with PAA added, and all grew (confirming the polymer inhibiting phage infection). The exception was *E. coli* BL21 (DE3) transformed with pT5T plasmid, encoding for DC-SIGN, where the growth rate was slightly faster with PAA. GFP expression was induced using IPTG (isopropyl thiogalactoside) and a negative control of no ITPG, with GFP expression confirmed by fluorescence spectroscopy. Addition of 10 mg·mL^–1^ of PAA had no noticeable impact on IPTG-induced GFP expression and did not induce leaky expression when no IPTG was added, confirming that it is a passive additive for this process, Figure 4.

**Figure 4:**
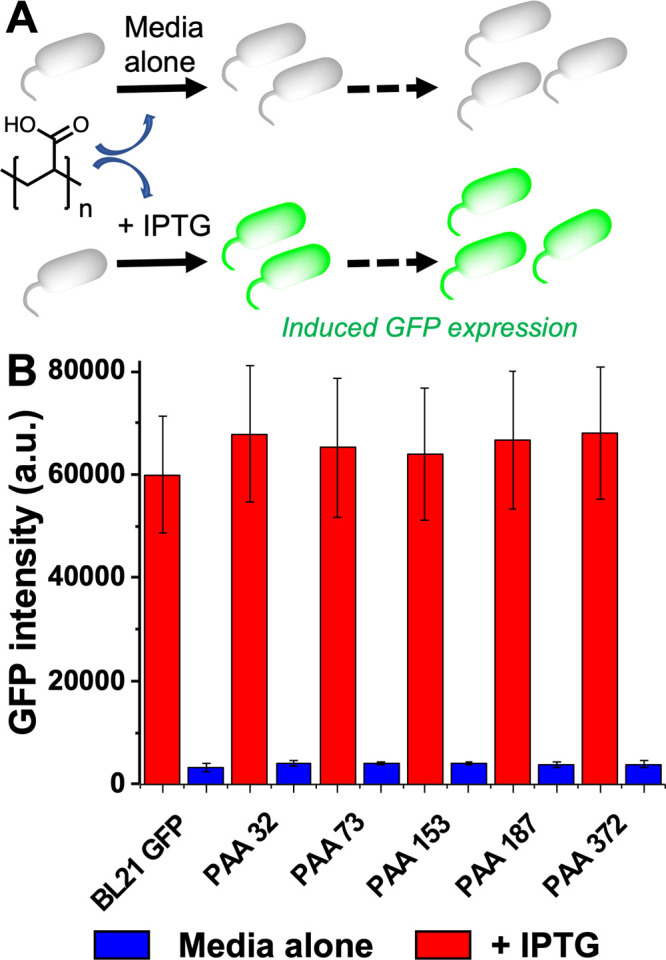
Recombinant expression of GFP in *E. coli* in the presence of PAA. (A) Schematic of experiment. (B) GFP production (fluorescence) after 3 h. BL21-GFP is the host strain alone. IPTG = isopropyl-β-d-thiogalactopyranoside, which induces expression. 0.4 mM. [polymer] = 10 mg·mL^–1^. Em. 528 nm; Ex 485 nm.

In conclusion, we demonstrate a simple, scalable solution to the problem of phage contamination in bacterial culture by using a polymeric additive. The low-cost, widely available water-soluble poly(acrylic acid) was identified to prevent phage infection during bacteria growth by simple addition into standard growth media. Exploration of a wide polymer-chemistry space revealed that uncharged polymers had no effect and that poly(methacrylic acid) was less active. Electron microscopy and dilution experiments support a virustatic mechanism of action rather than virucidal, suggesting the polymer reversibly interacts with the phage. The polymer does not impact *E. coli* growth, and in a recombinant protein (IPTG induction of GFP) system, the polymer did not affect the expression. These results are of crucial importance for a range of fields, since phage infection is a major problem in all biotechnology and microbiology research and manufacturing facilities, leading to closures and financial and scientific losses. By simple addition of this polymer additive, phage infection can be mitigated, reducing the need for shut down, fumigation, and other time-consuming and costly actions. Furthermore, this additive may help improve scientific quality by preventing accidental phage infection.

## Supporting Information Available

Full synthetic and characterization details alongside all bacteria/phage growth curves are also included (PDF)
