# Supplementary material for: Anionic Synthetic Polymers Prevent Bacteriophage Infection
Source: J Am Chem Soc. 2023 Apr 17;145(16):8794–9. doi: 10.1021/jacs.3c01874 (PMC10141250; doi:10.1021/jacs.3c01874)
Supplement: Supplementary file 1 — ja3c01874_si_001.pdf [file ja3c01874_si_001.pdf]

Supporting Information

"Anionic Synthetic Polymers Prevent Bacteriophage Infection''

Huba L Marton,[a] Peter Kilbride[d], Ashfaq Ahmad[b], Antonia P. Sagona,*[c] and Matthew I. Gibson*[a,b]

[a] Department of Chemistry, University of Warwick, Gibbet Hill Road, CV4 7AL, Coventry, UK;

[b] Division of Biomedical Sciences, Warwick Medical School, University of Warwick, Gibbet Hill Road, CV4 7AL, Coventry, UK;

[c] School of Life Sciences, University of Warwick, UK, CV4 7A;

[d] Asymptote, Cytiva, Chivers Way, Cambridge CB24 9BZ, U.K.

*<u>*Corresponding Authors*</u>: m.i.gibson@warwick.ac.uk (M.I.G.), A.Sagona@warwick.ac.uk (APS)

Experimental Section

Materials

All chemicals were used as supplied unless stated otherwise. 1-Dodecanethiol (≥98%), 2-bromo-2-methylpropionic acid (98%), carbon disulfide (anhydrous, ≥99%), tripotassium phosphate (≥98%), pentafluorophenol (≥99%), hydrochloric acid (reagent grade), dichloromethane (≥99.8%), ethyl acetate (99.8%), *N,N*-dimethylformamide (99%, DMF), tetrahydrofuran (≥99.9%, THF), diethyl ether (≥ 99.7%), n-hexane (reagent grade), petroleum ether (boiling point 40-60 °C) silica gel, 4,4'-azidobis(4-cyanovaleric acid) (≥75%, ACVA), poly(ethylene glycol) methacrylate (M_n 360, PEGMA), methacrylic acid (99%, MA), acrylic acid (99%, AA), *N*-hydroxyethyl acrylamide (97%, HEA), *N*-isopropyl acrylamide (97%, NIPAM), 4-cyano-4-(phenylcarbonothioylthio)pentanoic acid, 2-(dodecylthiocarbonothioylthio)-2-methylpropionic acid 3-azido-1-propanol ester (98%), 2-cyano-2-propyl dodecyl trithiocarbonate (97%), 4-cyano-4-[(dodecylsulfanylthiocarbonyl)sulfanyl]pentanoic acid (97%), chloroform-d_1 (99.8%), methanol-d_4 (≥99.8%), deuterium oxide (99.9%), agarose, lysogeny broth (LB), poly(ethylene glycol) PEG (M_n 4,000), poly(sodium 4-styrene sulfonate) (M_w 70,000) were all purchased from Sigma-Aldrich (Merck). Caesium chloride, magnesium sulfate, magnesium sulfate heptahydrate, sodium chloride, sodium hydrogen carbonate, methanol (reagent grade), acetone (reagent grade), acetic acid (glacial), and PEG (Mn 8,000), were purchased from Fisher Scientific. Glycerol was purchased from Scientific Laboratory Supplies (SLS). *N*-(3-Dimethylaminopropyl)-*N'*-ethylcarbodiimide hydrochloride (EDC.HCl) was purchased from Carbosynth. Tris-HCl (24.2 g.L^{-1} Tris, 80 g.L^{-1} NaCl), kanamycin (50 μg.mL^{-1}) and ampicillin (100 μg.mL^{-1}) were prepared by media preparation facility in the School of Life Sciences at the University of Warwick. SM-I buffer (1 M NaCl, 8 mM $MgSO_4.7H_2O$, 22.5 mM Tris-HCl

pH 7.5) and SM-II buffer (100 mM NaCl, 8 mM $MgSO_4.7H_2O$, 22.5 mM Tris-HCl pH 7.5) were both prepared in house.

Physical and Analytical Methods

NMR spectroscopy.

Proton (^{1}H-NMR) nuclear magnetic resonance spectra were recorded at 300 MHz or 400 MHz on a Bruker DPX-300 or DPX-400 spectrometer respectively, with methanol-d4 (CD_3OD), chloroform-d ($CDCl_3$), dimethyl sulfoxide-d6 ($(CD_3)_2SO$) and deuterium oxide (D_2O) as the solvents. Chemical shifts of protons are reported as δ in parts per million (ppm). Chemical shifts of protons are reported as δ in parts per million (ppm) and are relative to tetramethylsilane (TMS) at δ = 0 ppm when using CDCl3 or solvent residual peak (CH3OH, δ = 3.31 ppm) for CD_3OD and ($(CH_3)_2SO$, δ = 2.54 ppm) for $(CD_3)_2SO$, (H_2O, δ = 4.78 ppm).

Fourier Transformed-Infrared (FT-IR) spectroscopy.

Fourier-Transform-Infrared (FT-IR) spectroscopy measurements were carried out in the range of 650 to 4000 cm^{-1} using a Cary 630 FT-IR spectrometer (Agilent).

Size exclusion chromatography (SEC) in DMF.

Size exclusion chromatography (SEC) analysis was performed on an Agilent Infinity II MDS instrument equipped with differential refractive index (DRI), viscometry (VS), dual angle light scatter (LS) and variable wavelength UV detectors. The system was equipped with 2 × PLgel Mixed D columns (300 × 7.5 mm) and a PLgel 5 µm guard column. The mobile phase used was DMF (HPLC grade) containing 5 mM NH_4BF_4 at 50 °C at a flow rate of 1.0 mL.min^{-1}. Poly(methyl methacrylate) (PMMA) standards (Agilent EasyVials) were used for calibration between 955,000 – 550 g.mol^{-1}. Analyte samples were filtered through a nylon membrane with 0.22 μm pore size before injection. Number average molecular weights (M_n), average molecular

weights (M_w) and dispersities ($Đ_M = M_w/M_n$) were determined by conventional calibration and universal calibration using Agilent GPC/SEC software.

Optical density measurements and corrections.

For the preliminary optical density measurements at 600 nm (OD_{600}) of the bacterial growth curves described below, a Fisher Scientific portable cell density meter, model 40 was used. The OD_{600} preliminary measurements of the green fluorescent protein (GFP) expression assays described below were performed on a Jenway 6300 benchtop visible spectrophotometer.

Dynamic Light Scattering.

Dynamic light scattering (DLS) measurements were performed on a Zetasizer ZS (Malvern Panalytical, Worcestershire, UK). Each measurement was carried out using a 4 mW He-Ne 633 nm laser module operating at 25 °C and an angle of 173° (back scattering). Data was analysed using Malvern DTS 7.03 software. Measurements were repeated in triplicate.

Synthesis of *2-(dodecylthiocarbonothioylthio)-2-methyl propionic acid (DMP)*

This was synthesised, according to a previously published method.[1] 1-dodecane thiol (2.00 g, 9.88 mmol) was added dropwise to stirring K_3PO_4 (2.10 g, 9.89 mmol) in acetone (30 mL) at RTP, leaving the mixture stirring for 25 minutes to form a white suspension. Carbon disulphide (2.05 g, 26.93 mmol) was then added, and mixture stirred for 10 minutes, to form a yellow solution. 2-bromo-2-methyl-propionic acid (1.5 g, 8.98 mmol) was added, and solution left to stir for 16 hours. Solvent was removed under vacuum. The crude product was dissolved in 1M HCl (100 mL) and extracted with DCM (2 × 100 mL). Organic layer was washed with water (200 mL) and brine (200 mL), then dried with $MgSO_4$ and filtered under gravity. The solvent from the filtrate was removed under vacuum. The crude product was purified using a silica column (40-60 PET : DCM : glacial acetic acid 75:24:1) and recrystallized in n-hexane to give a yellow solid (32%). ^{1}H NMR (300 MHz, $CDCl_3$) δ = 3.30 (2H,t, SCH_2**CH_2**), 1.75 (6H, s,

C(**CH_3**)**$_2$**), 1.69 (2H, qn, S**CH_2**), 1.46 - 1.22 (18H, m,(**CH_2**)$_9$$CH_3$), 0.93 - 0.87 (3H, m, **CH_3**). m/z calculated as 364.16; found for ESI $[M+H]^+$ 365.1 and $[M+Na]^+$ 387.1. FTIR (cm^{-1}) – 2956, 2917 & 2848 (methyl and methylene), 1702 (ester C=O), 1459, 1437 & 1413 (methyl and methylene), 1280 (C(**CH_3**)**$_2$**), 1064 (S-C(S)-S).

Synthesis of Pentafluorophenyl-2-dodecylthiocarbonothioylthio)-2-methylpropanoate (PFP-DMP)

PFP-DMP was kindly provided by Dr Alexander Baker, synthesised, according to a previously published procedure.[1] DMP (4.06 g, 11.13 mmol), EDC (3.65 g , 19.04 mmol) and DMAP (2.30 g, 18.82 mmol) were dissolved in DCM (160 mL) and degassed with nitrogen for 30 minutes. Pentafluorophenol (7.28 g, 39.55 mmol) in DCM (20 mL) was added and the mixture stirred for 18 hours at RTP. The organic layer was washed with 3 M HCl (200mL), 1 M $NaHCO_3$ (200 mL) and 0.5 M NaCl (200 mL), followed by drying with $MgSO_4$ and gravity filtration. The solvent was then removed from the filtrate under vacuum. The crude product was recrystallised in ethyl acetate (or hexane) overnight at -8 °C and dried to give yellow

crystals (90.9%). ^{1}H (300 MHz, $CDCl_3$) δ = 3.31 (2H,t, J 7.5, SCH_2**CH_2**), 1.86 (6H, s, C(**CH_3**)**$_2$**), 1.69 (2H, qn, J 7.5, S**CH_2**), 1.48 - 1.16 (18H, m, **(CH_2)$_9$**CH3), 0.94 - 0.82 (3H, m, CH_3). dC (300 MHz, CDC13) 220.06 (1C, SC(S)S), 169.71 (1C, C(O)), 143.13 (2C, meta C), 139.79 (1C, ipso C), 139.61 (1C, para C), 136.30 (2C, Ortho C), 55.50 (1C, C(CH3)2), 37.26 (1C, SCH_2), 32.03 - 22.81 (10C, $SCH_2(CH_2)_{10}$), 25.37 (2C, $C(CH_3)_2$), 14.11 (1C, CH_2CH_3). ^{19}F (300 MHz, CDC13) δ -151.44- -151.61- (2F, m, OCC2H2C2H2CH), -148.50 (1F, t, J 21.5, OCC2H2C2H2CH), -162.23 - -162.47 (2F, m, OCC2H2C2H2CH). m/z calculated as 530.14; found for ESI $[M+Na]^+$ 553.3 and $[M+CH_3CN+Na]^+$ 593.5. FTIR (cm^{-1}) – 2955.8, 2916.6 & 2849.5 (methyl and methylene), 1701.5 (ester C=O), 1518.9 (aromatic C=C or C-F), 1459.3, 1436.9 & 1412.7 (methyl and methylene), 1280.3 (C(CH3)2), 1067.9 (S-C(S)-S).

Synthesis of Poly(poly(ethylene glycol) methacrylate)) (PPEGMA)

As a representative example, poly(ethylene glycol) methacrylate (M_n 360) (1.29 g, 3.58 mmol, 50 eq), 4-cyano-4-(phenylcarbonothioylthio) pentanoic acid (0.02 g, 0.07 mmol, 1 eq), 4,4'-azidobis(4-cyanovaleric acid) (0.004 g, 0.014 mmol, 0.2 eq), DMF (6.6 mL) as solvent were added to a glass vial. A stirrer bar was added, the vial sealed with a Suba seal, solution bubbled with nitrogen for 20 minutes, and reaction left stirring at 60 ºC overnight, after taking a small sample to determine conversion. Next day, the reaction was stopped by submerging the glass vial in liquid nitrogen and exposing solution to air. Crude polymer was precipitated from diethyl ether (2 × 50 mL) and dried under vacuum. The resulting polymer was analysed by ^{1}H NMR, and SEC. Representative characterization data for $PPEGMA_{50}$: ^{1}H NMR (400 MHz, DMSO-d_6): δ: 7.87 – 7.43 (5H, m, Ar), 4.61 – 4.47 (3H, s, CNC**(CH_3)**CH2CH2), 4.06 – 3.96 (4H, m, CNC(CH_3)**CH2CH2**), 3.67 – 3.46 (4H, m, COO**CH2CH2**OCH_3), 3.38 – 3.28 (3H, s, COOCH2CH2O**CH_3**) 2.10 – 1.52 (2H, m, SCSC(CH_3)**CH_2**), 1.02 – 0.62 (3H, m, SCSC(**CH_3**)CH_2). M_n^{SEC} (DMF) = 27700 g.mol^{-1}, $Đ_M$ = 1.65.

Synthesis of Poly(methacrylic acid) (PMA)

As a representative example, methacrylic acid (0.31 g, 3.58 mmol, 100 eq), 4-cyano-4-(phenylcarbonothioylthio) pentanoic acid (0.01 g, 0.036 mmol, 1 eq), 4,4'-azidobis(4-cyanovaleric acid) (0.002 g, 0.0072 mmol, 0.2 eq), methanol (1.6 mL) as solvent were added to a glass vial. A stirrer bar was added, the vial sealed with a Suba seal, solution degassed with nitrogen for 20 minutes, a small sample taken to determine conversion and reaction left stirring at 60 ºC overnight. Next day, the reaction was stopped by submerging the glass vial in liquid nitrogen and exposing solution to air. Crude polymer was precipitated from diethyl ether (2 × 50 mL) and dried under vacuum. The resulting polymer was analysed by ^{1}H NMR, and SEC. Representative characterization data for PMA_{100}: ^{1}H NMR (400 MHz, CD_3OD): δ 7.93 – 7.34

(5H, m, Ar), 3.78 – 3.58 (3H, m, C(**CH_3)**$CNCH_2CH_2$), 2.29 – 1.74 (2H, m, $SCSC(CH_3)(COOH)$**CH_2**), 1.72 – 1.47 (2H, m, $COOH$**CH_2**CH_2)), 1.46 – 1.29 (2H, m, $COOHCH_2$**CH_2**), 1.15 – 1.00 (3H, m, SCSC(**CH_3)**(COOH)CH_2). M_n^{SEC} (DMF) = 18400 g.mol^{-1}, $Đ_M$ = 1.21.

Synthesis of Poly(acrylic acid) (PAA)

As a representative example, acrylic acid (1.98 g, 27.46 mmol, 100 eq), DMP (0.1 g, 0.27 mmol, 1 eq), 4,4'-azidobis(4-cyanovaleric acid) (0.015 g, 0.055 mmol, 0.2 eq), methanol (10.5 mL) as solvent were added to a glass vial. A stirrer bar was added, the vial sealed with a Suba seal, solution degassed with nitrogen for 20 minutes, a small sample taken to determine conversion and reaction left stirring at 60 ºC overnight. Next day, the reaction was stopped by submerging the glass vial in liquid nitrogen and exposing solution to air. Crude polymer was precipitated from diethyl ether (2 × 50 mL) and dried under vacuum. The resulting polymer was analysed by ^{1}H NMR, and SEC. PAA used for inhibition experiments, after initial screening) was further dialysed to remove residual solvent. Representative characterization data for PAA_{100}: ^{1}H NMR (400 MHz, CD_3OD): δ 3.45 – 3.38 (4H, m, C(S)S**$(CH_2)_2$**CH_2CH_2), 2.78 – 2.66 (6H, m, COOHC**$(CH_3)_2$**), 2.64 – 2.25 (1H, m, S**CH**(COOH)CH_2) , 2.22 – 1.48 (2H, m, SCH(COOH)**CH_2**) 1.46 – 1.28 (18H, m, CH_3**$(CH_2)_9$**CH_2), 0.96 – 0.89 (3H, t, **CH_3**$(CH_2)_{11}$SCS). M_n^{SEC} (DMF) = 11000 g.mol^{-1}, $Đ_M$ = 1.28.

Alternative chain transfer agent used was 2-Cyano-2-propyl dodecyl trithiocarbonate.

Synthesis of Poly(N-hydroxyethyl acrylamide) (PHEA)

As a representative example, *N*-hydroxyethyl acrylamide (1.00 g, 8.68 mmol, 100 eq), 2-cyano-2-propyl dodecyl trithiocarbonate (0.03 g, 0.087 mmol, 1 eq), 4,4'-azidobis(4-cyanovaleric acid) (ACVA) (0.0049 g, 0.018 mmol, 0.2 eq), methanol (5.2 mL) as solvent were added to a glass vial. A stirrer bar was added, the vial sealed with a Suba seal, solution degassed with

nitrogen for 20 minutes, a small sample taken to determine conversion and reaction left stirring at 60 ºC overnight. Next day, the reaction was stopped by submerging the glass vial in liquid nitrogen and exposing solution to air. Crude polymer was precipitated from diethyl ether (2 × 50 mL) and dried under vacuum. The resulting polymer was analysed by ^{1}H NMR, and SEC. Representative characterization data for $PHEA_{100}$: ^{1}H NMR (400 MHz, CD_3OD): δ 8.16 – 7.94 (1H, m, CO**NH**CH_2CH_2OH), 3.81 – 3.52 (2H, m, CONHCH_2**CH_2**OH), 3.41 – 3.06 (2H, t, CONH**CH_2**CH_2OH), 2.4 – 1.77 (1H, m, S**CH**(CONHCH_2CH_2OH)CH_2), 1.77 – 1.30 (2H, m, SCH(CONHCH_2CH_2OH)**CH_2**), 1.30 – 1.20 (6H, s, CNC**$(CH_3)_2$**), 0.79 – 0.69 (3H, t, **CH_3**$(CH_2)_{11}S$) (CH_2 protons from the 12 carbon end of the RAFT agent were overlapped by two backbone CH_2). M_n^{SEC} (DMF) = 8900 g.mol^{-1}, $Đ_M$ = 1.36.

Alternative chain-transfer agent used was 4-cyano-4-[(dodecylsulfanylthiocarbonyl)sulfanyl] pentanoic acid, included in the table below.

Addition Synthesis of Poly(N-hydroxyethyl acrylamide) (PHEA)

Alternatively, a photo-polymerization was also included for the synthesis of PHEA polymers, which were conducted later in the project.[2] As a representative example, *N*-hydroxyethyl acrylamide (1.08 g, 9.42 mmol, 50 eq), PFP-DMP (0.1 g, 0.19 mmol, 1 eq), and methanol (5.9 mL) as solvent were added to a glass vial. A stirrer bar was added, the vial sealed with a Suba seal, solution degassed with nitrogen for 20 minutes, a small sample taken to determine conversion and reaction left stirring at RTP under blue light (460 nm) for 5 hours. Afterwards, the reaction was stopped by submerging the glass vial in liquid nitrogen and exposing solution to air. Crude polymer was precipitated from diethyl ether (2 × 50 mL) and dried under vacuum. The resulting polymer was analysed by ^{1}H NMR, and SEC. Representative characterization data for $PHEA_{50}$: ^{1}H NMR (400 MHz, CD_3OD): δ 8.28 – 7.90 (1H, m, CO**NH**CH_2CH_2OH), 3.95 – 3.45 (2H, t, CONHCH_2**CH_2**OH), 3.44 – 3.22 (2H, t, CONH**CH_2**CH_2OH), 2.29 – 1.92

(1H, m, S**CH**(CONHCH$_2$CH$_2$OH)CH$_2$), 1.90 – 1.45 (2H, m, SCH(CONHCH$_2$CH$_2$OH)**CH$_2$**), 1.34 – 1.26 (14H, t, CH$_3$CH$_2$**(CH$_2$)$_7$**), 0.99 – 0.85 (3H, t, **CH$_3$**(CH$_2$)$_2$S) (unaccounted CH$_2$ protons from the 12 carbon end of the RAFT agent, and the 2 × CH$_3$ near PFP end of RAFT agent were overlapped by backbone proton peaks). M_n^{SEC} (DMF) = 4500 g.mol^{-1}, $Đ_M$ = 1.37.

Alternative chain-transfer agent used was 4-cyano-4-[(dodecylsulfanylthiocarbonyl)sulfanyl] pentanoic acid, included in the table below.

Synthesis of Poly(N-isopropyl acrylamide) (PNIPAM)

As a representative example, *N*-isopropyl acrylamide (1.55 g, 13.73 mmol, 100 eq), DMP (0.05 g, 0.14 mmol, 1 eq), 4,4'-azidobis(4-cyanovaleric acid) (ACVA) (0.0077 g, 0.0028 mmol, 0.2 eq), THF (8.1 mL) as solvent were added to a glass vial. A stirrer bar was added, the vial sealed with a Suba seal, solution degassed with nitrogen for 20 minutes, a small sample taken to determine conversion and reaction left stirring at 60 ºC overnight. Next day, the reaction was stopped by submerging the glass vial in liquid nitrogen and exposing solution to air. Crude polymer was precipitated from diethyl ether (2 × 50 mL) and dried under vacuum. The resulting polymer was analysed by ^{1}H NMR, and SEC. Representative characterization data for PNIPAM$_{100}$: ^{1}H NMR (400 MHz, CD$_3$OD): δ 8.21 – 7.82 (1H, m, CO**NH**CH(CH$_3$)$_2$), 4.13 – 3.85 (1H, m, CONH**CH**(CH$_3$)$_2$), 3.80 – 3.70 (2H, t, S**CH$_2$**(CH)$_{10}$CH$_3$), 2.33 – 1.93 (1H, m, S**CH**(CONHCH(CH$_3$)$_2$)CH$_2$), 1.92 – 1.86 (6H, t, C**(CH$_3$)$_2$**COOH), 1.83 – 1.38 (2H, m, SCH(CONHCH(CH$_3$)$_2$)**CH$_2$**), 1.38 – 1.28 (10H, t, CH$_3$**(CH$_2$)$_5$**CH$_2$), 1.27 – 1.06 (6H, s, SCH(CONHCH**(CH$_3$)$_2$**)CH$_2$), 0.95 – 0.89 (3H, t, **CH$_3$**(CH$_2$)$_{11}$S) (unaccounted CH$_2$ of the 12 carbon end of the RAFT agents were overlapped by backbone CH$_2$ protons). M_n^{SEC} (DMF) = 10800 g.mol^{-1}, $Đ_M$ = 1.22.

Alternative chain transfer agent used was 2-Cyano-2-propyl dodecyl trithiocarbonate.

Table S1. Polymer Characterization

Code	[M]:[CTA] (-)	$Mn_{,SEC}$ (g.mol^{-1})	Đ (-)	DP (-)
**PPEGMA 35**	25	12600	1.36	35
**PPEGMA 40**	25	14400	1.10	40
**PPEGMA 57**	50	20500	1.09	57
**PPEGMA 77**	50	27700	1.65	77
**PPEGMA 102**	100	36800	1.10	102
**PPEGMA 134**	100	48300	2.75	134
**PPEGMA 222**	300	79800	2.04	222
**PPEGMA 382**	500	137600	4.15	382
**PMA 89**	25	7700	1.17	89
**PMA 155**	50	13300	1.11	155
**PMA 155**	50	13200	1.19	155
**PMA 208**	100	18000	1.16	208
**PMA 213**	100	18400	1.21	213
**PMA 278**	200	24000	1.14	278
**PMA 329**	200	28200	1.21	329
**PMA 406**	500	35000	1.27	406
**PAA 73**	25	5300	1.18	73
**PAA 32**	50	2274	1.10	32
**PAA 153**	100	11000	1.28	153
**PAA 187**	200	13500	1.26	187
**PAA 372**	500	26800	1.34	372
**PHEA 30**	25	3500	1.19	30
**PHEA 45**	25	5200	1.30	45
**PHEA 39**	50	4500	1.37	39
**PHEA 86**	50	9900	1.22	86
**PHEA 78**	100	8900	1.36	78
**PHEA 189**	75	21800	1.12	189
**PHEA 199**	200	22900	1.31	199
**PHEA 227**	150	26100	1.63	227
**PHEA 279**	500	32100	1.43	279
**PNIPAM 37**	25	4100	1.18	37
**PNIPAM 35**	50	3900	1.22	35
**PNIPAM 96**	100	10800	1.22	96
**PNIPAM 31**	200	3500	1.27	31
**PNIPAM 119**	200	13500	1.52	119
**PNIPAM 161**	500	18200	1.52	161
**PNIPAM 284**	500	32100	1.20	284

Table S2. Polymerization Components

Code	CTA	Initiator	Solvent	Polymerization type
**PPEGMA 35**	1	ACVA	DMF	Thermal
**PPEGMA 40**	1	ACVA	DMF	Thermal
**PPEGMA 57**	1	ACVA	DMF	Thermal
**PPEGMA 77**	1	ACVA	DMF	Thermal
**PPEGMA 102**	1	ACVA	DMF	Thermal
**PPEGMA 134**	1	ACVA	DMF	Thermal
**PPEGMA 222**	1	ACVA	DMF	Thermal
**PPEGMA 382**	1	ACVA	DMF	Thermal
**PMA 89**	1	ACVA	MeOH	Thermal
**PMA 154**	1	ACVA	MeOH	Thermal
**PMA 155**	1	ACVA	MeOH	Thermal
**PMA 208**	1	ACVA	MeOH	Thermal
**PMA 213**	1	ACVA	MeOH	Thermal
**PMA 278**	1	ACVA	MeOH	Thermal
**PMA 329**	1	ACVA	MeOH	Thermal
**PMA 406**	1	ACVA	MeOH	Thermal
**PAA 73**	5	ACVA	MeOH	Thermal
**PAA 32**	2	ACVA	MeOH	Thermal
**PAA 153**	5	ACVA	MeOH	Thermal
**PAA 187**	2	ACVA	MeOH	Thermal
**PAA 372**	2	ACVA	MeOH	Thermal
**PHEA 30**	6	-	MeOH	Photo
**PHEA 45**	3	ACVA	MeOH	Thermal
**PHEA 39**	6	-	MeOH	Photo
**PHEA 86**	4	ACVA	MeOH	Thermal
**PHEA 78**	3	ACVA	MeOH	Thermal
**PHEA 189**	6	-	MeOH	Photo
**PHEA 199**	4	ACVA	MeOH	Thermal
**PHEA 227**	6	-	MeOH	Photo
**PHEA 279**	4	ACVA	MeOH	Thermal
**PNIPAM 37**	5	ACVA	THF	Thermal
**PNIPAM 35**	4	ACVA	THF	Thermal
**PNIPAM 96**	5	ACVA	THF	Thermal
**PNIPAM 31**	4	ACVA	THF	Thermal
**PNIPAM 119**	5	ACVA	THF	Thermal
**PNIPAM 161**	5	ACVA	THF	Thermal
**PNIPAM 284**	4	ACVA	THF	Thermal

Chain transfer agents (CTA).

CTA 1 4-Cyano-4-(phenylcarbonothioylthio)pentanoic acid

CTA 2 2-(Dodecylthiocarbonothioylthio)-2-methylpropionic acid 3-azido-1-propanol ester

CTA 3 2-Cyano-2-propyl dodecyl trithiocarbonate

CTA 4 4-Cyano-4-[(dodecylsulfanylthiocarbonyl)sulfanyl]pentanoic acid

CTA 5 2-(Dodecylthiocarbonothioylthio)-2-methyl propionic acid (DMP)

CTA 6 Pentafluorophenyl-2-dodecylthiocarbonothioylthio)-2-methylpropanoate (PFP-DMP)

Biological Methods

Viral enrichment – propagation of K1F-GFP, K1E, K15, T7 and T4 bacteriophages.

To propagate the library of bacteriophage isolates, *Escherichia coli* (*E. coli)* EV36 host for K1F-GFP, K1E and K1-5 and *E. coli* AB1157 host for T7 and T4 phages, were grown overnight in lysogeny broth (LB) (Sigma-Aldrich: Lennox – 10 g.L^{-1} tryptone, 5 g.L^{-1} yeast extract, 5 g.L^{-1} NaCl) at 37 °C and 130 rpm. *E. coli* AB1157 was used for the propagation of T7 and T7 phages, whereas the host for the assays described below for these phages was *E. coli* K-12 (MG1655 cells). The following morning, 1 mL of the overnight liquid cultures were used to inoculate 50 mL of fresh LB, for each phage separately. This newly inoculated LB was incubated at 37 °C and 130 rpm until an OD_{600} (optical density at 600 nm) of 0.3 was reached. At this point, 100 - 300 µL of bacteriophage stock (depending on previous stock's concentration) was added to each corresponding flask and the samples incubated for a further four hours. The bacterial debris of *E. coli* EV36 and AB1157 were pelleted by centrifugation at 3220 g for 10 min before passing the supernatants through a 0.2 µm pore size membrane filter. Five prepared phage stocks in LB were stored at 4 °C.

Caesium chloride purification of bacteriophages.

For the purification of all bacteriophages the previously described propagation assay was scaled up to 250 mL per sample by transferring the supernatant into LB media. Sodium chloride was added to each sample to achieve a final concentration of 1 M. After 1 hour incubation on ice, each phage sample was centrifuged at 3220 g and the supernatant filtered through at 0.2 µm pore size membrane before adding PEG 8000 to a final concentration of 10% w/v. Phage samples were left overnight at 4 °C, before centrifugation at 25 000 g for one hour. Phage pellets were resuspended in 6-7 mL SM buffer I and passed through a 0.2 µm pore size membrane, before undergoing concentration and purification in a CsCl gradient for 20 hours

at 150,000 g and 4 °C. Following the centrifugation, phages were concentrated into a band, shown in figure S1, for K1E and K1-5 phages.

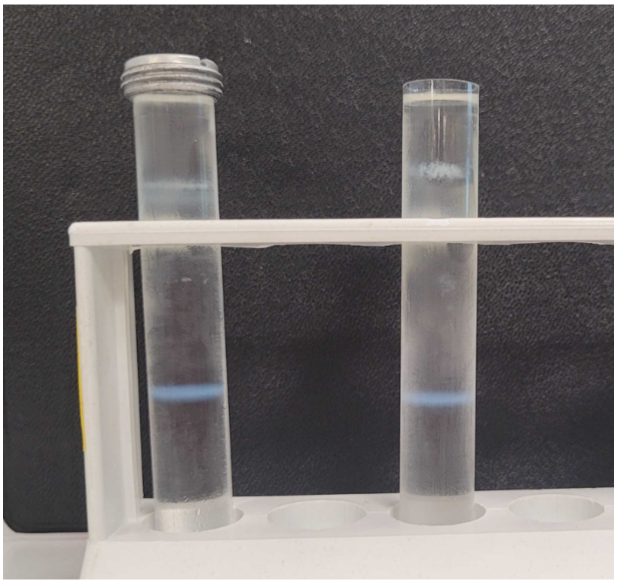

Figure S1. Caesium chloride gradient band formation of K1E (left) and K1-5 (right) phages.

The band was syringe extracted, and first dialysed in SM buffer I and twice dialysed in SM buffer II to remove the CsCl. Purified phage samples were stored at 4 °C.

***E. coli* growth curves – high throughput testing of polymer library.**

E. coli EV36 and K-12 were separately grown in a FLUOstar Omega microplate reader at 37 °C taking measurements of the optical density (OD_{600} or Abs_{600}) every five minutes over a 24 hour period. Bacteriophages were incubated in polymer (10 $mg.mL^{-1}$) dissolved in SM-II buffer solutions overnight, with a starting concentration of at least 1×10^6 $PFU.mL^{-1}$, prior to the assay. Bacteria hosts with a final concentration of 1×10^6 $CFU.mL^{-1}$ (OD_{600} 0.001) were transferred to a 96-well plate and grown for four hours at 37 °C with shaking to reach the log phase. During the log phase, the overnight polymer incubated phage aliquots were added to each corresponding well of the plate including 1% v/v or 10% v/v Chemgene surface disinfectant as a positive control. SM-II buffer and PEG 4000 in SM-II buffer incubated phages were used as additional controls, at matching concentrations to polymer incubated aliquots. All

samples were grown shaking in lysogeny broth (LB) media in a total volume of 200 μL, including LB blank wells. Data was obtained and blank corrected using the MARS analysis software (Version 5.10). The growth curves were carried out in biological triplicate unless stated otherwise.

***E. coli* poly(acrylic acid) dose response growth curves.**

Similarly, to the previously described assay, EV36 and K-12 strains of *E. coli* were grown in the FLUOstar Omega microplate reader at 37 °C taking measurements of the optical density (OD_{600}) every five minutes over 24 hours. Each of the five molecular weight (M_W) solutions of poly(acrylic acid) were serially diluted from 10 $mg.mL^{-1}$ to either 0.3125 $mg.mL^{-1}$ or 0.15625 $mg.mL^{-1}$ (depending on phage used). Then all five bacteriophages were separately incubated for one hour in each serial dilution of PAA in SM-II with a concentration of at least 1×10^6 $PFU.mL^{-1}$, prior to the assay. A key aspect of the preparation was to ensure the PAA dilutions were performed before adding the phages, not after. Bacteria hosts prepared with a final concentration of 1×10^6 $CFU.mL^{-1}$ were transferred to a 96-well plate and grown for four hours at 37 °C with shaking to reach the log phase. During the log phase, the phage in PAA incubated aliquots were added to each corresponding well of the plate. Additional 1% v/v Chemgene surface disinfectant, PEG 4000 incubated phages and SM-II incubated phages (matching PAA sample concentrations) were included as extra controls. Every sample was grown with shaking in lysogeny broth (LB) media in a total volume of 200 μL, including LB blank wells. The data was blank corrected and obtained using the MARS analysis software (Version 5.10). Each dose response growth curve was carried out in biological triplicate unless stated otherwise.

Plaque assay – quantification of bacteriophage concentration.

Bacteriophage titres for K1F-GFP, K1E, K1-5, T7 and T4 phages were determined via a soft agar plaque assay, using 0.7% agar lysogeny broth agar (LBA) overlay.[3] Similarly to the growth curve assay, the bacteriophages were incubated in SM-II buffer dissolved polymer solutions overnight with a starting concentration of at least 1 × 10^6 PFU.mL^{-1}. The following day, 100 µL aliquot of serially diluted overnight incubated phages were further incubated with an equal volume of bacteria host cell lawn (~ 1 × 10^8 CFU.mL^{-1} (colony forming units)) at room temperature for 15 minutes before the addition of 3 mL liquid agar overlay (0.7 % agar) and pouring over a solid 1.5% agar LBA plate. After an overnight incubation at 37 °C, the individually distinct zones of clearance on plates (plaques) were enumerated and quantified as PFU.mL^{-1} (plaque forming units) taking the serial dilution into account. The assay was carried out as one biological replicate with two technical replicates per polymer sample, as the primary aim was to determine the presence or absence of plaques post polymer incubation.

Electron microscopy of phages K1F-GFP and T4.

The K1F-GFP and T4 phage lysates in SM-II, with a concentration ~ 1 × 10^{10} PFU.mL^{-1} were initially diluted to concentration 1 × 10^9 PFU.mL^{-1}. After the addition of PAA 153 (used as the poly(acrylic acid) test) phage aliquots with final concentration ~ 1 × 10^8 PFU.mL^{-1} were incubated in PAA 153 or SM-II without polymer for one hour. PAA 153 incubated phages were further diluted to concentration ~ 5 × 10^7 PFU.mL^{-1} due to high phage density observed initially. Bacteriophages were then incubated on a Formvar/carbon-coated grid (EMResolutions) which was previously glow discharged for 1 minute. After 10 minutes, the grid was washed three times with 1% phosphate buffered saline (PBS), followed by 4 minute incubation with 2 % uranyl acetate staining (negative staining). All four grids were imaged using a Jeol 2100Plus TEM microscope fitted with a Gatan OneView IS camera at 200 kV.

K1F-GFP and T4 phages incubated in SM-II control images were obtained at 40× magnification, whereas the PAA 153 incubated K1F-GFP and T4 images were obtained at 30× and 60× magnifications, respectively. The images were then processed using Fiji (ImageJ) software.

***E. coli* recombinant GFP expression assay.**

A pWALDO plasmid encoding for the hexahistidine-tagged green fluorescent protein (GFP) was kindly provided by Elizabeth Fullam (University of Warwick, Coventry, UK). A pT5T plasmid encoding for the human lectin i.e., DC-SIGN (Dendritic Cell-Specific Intercellular adhesion molecule-3-Grabbing Non-integrin), was kindly provided by Daniel Mitchell (University of Warwick, Coventry, UK) and used as a negative control. Wild-type untransformed BL21 (DE3) cells lacking any plasmid were also used as negative control. The plasmids were transformed into competent *Escherichia coli* BL21 (DE3) cells (New England Biolabs). Overnight cultures were prepared by inoculating 10 mL of LB medium with a colony and grown overnight at 37 °C, and 165 rpm, using appropriate antibiotics (50 μg.mL^{-1} of kanamycin for pWALDO, and 50 μg.mL^{-1} of ampicillin for pT5T). The overnight culture for the wild type untransformed BL21 (DE3) cells was grown in the absence of antibiotics. Same protocol was used for all three *E. coli* BL21 (DE3) strains from here onwards. The following day, 10 mL of fresh LB-medium (supplemented with appropriate antibiotics as mentioned above) was inoculated with 20 μL of the overnight culture. These were grown at 37 °C under continuous shaking at 165 rpm until OD_{600} of 0.25 – 0.27 was reached (around 4 hours). After OD corrections (where necessary) the cultures were cooled to room temperature before adding isopropyl β-D-1-thiogalactopyranoside (IPTG) to a final concentration of 0.4 mM. The cultures were then transferred to their respective wells in the 96-well plate. For the poly(acrylic acid) samples, additional 20 μL of 10 mg.mL^{-1} aliquots of all five molecular weights were added, post IPTG addition. The recombinant GFP intensity was measured using a SynergyHT multi-

mode microplate reader (BioTek UK, Bedfordshire, UK), using 485 nm excitation and 528 nm emission wavelengths. Fluorescence gain of the detector was adjusted to the *E. coli* BL21 (DE3) GFP expressing non-polymer wells, with a scale value of 20,000 (scaled to high intensity wells). However, due to overexpression of GFP leading to fluorescence oversaturation the intensity was measured for a maximum of three hours. Data was blank corrected and obtained using the BioTek Gen5 Microplate Reader and Imager Software (Agilent, Version 3.0). The assay was carried out in biological triplicate.

Bacteriophage Virustatic- versus Virucidal- assay.

Antiviral assays for K1F-GFP and T4 bacteriophages were conducted based on previous binding mechanism determination studies[4,5] to compare the mechanism of inhibition of the two polymers (PAA and PMA), that is, virucidal (irreversible) or virustatic (reversible). Briefly, the bacteriophages were incubated in 10 mg.mL^{-1} of PAA for 24 hours and 20 mg.mL^{-1} of PMA for 72-hours as a total volume of 200 µL at 4 °C, with a final concentration of 1.28 × 10^{10} PFU.mL^{-1} (K1F-GFP) and 6.5 × 10^{10} PFU.mL^{-1} (T4). K1F-GFP and T4 were chosen as two phage representatives with varied morphologies. Using the previously described growth curve assay, host cultures for K1F-GFP (*E. coli* EV36) and T4 (*E. coli* K-12) were grown from a starting concentration of 1 × 10^{6} CFU.mL^{-1} for 4 hours at 37°C to reach log phase. To assess any loss of inhibition, 10-fold serial dilutions (washes) was carried out four times, before adding the phages to corresponding host wells, at the log phase. In addition, 10% v/v Chemgene was added as an internal positive control. For each serial dilution a phage control was included with matching phage titre (PFU.mL^{-1}) to exclude any variation in infectivity due to reduced number of phages in the culture. All samples were grown in a total of 200 µL LB media with LB blanks included. The data was obtained and blank corrected using the MARS analysis software (Version 5.10).

Post-phage infection inhibition assay.

To investigate the 'therapeutic' (ability to clear an established phage infection) effect of poly(acrylic acid) and poly(methacrylic acid) post-infection polymer-mediated phage inhibition assays were carried out, using the previously described growth curve assay. Briefly, the bacteriophages were initially added to corresponding host liquid cultures which reached log (exponential) phase in the 96-well plate, which created the 'infection scenario' shortly followed by the addition of the polymers. Bacteriophages K1F-GFP (final concentration of 1.28×10^{10} PFU.mL^{-1}) and T4 (final concentration of 6.5×10^{10} PFU.mL^{-1}) were added to 160 µL host liquid culture, as 20 µL aliquots, followed by the further addition of 20 µL PAA or PMA aliquots of either 10 or 20 mg.mL^{-1}, leading to a total well volume of 200 µL. Similarly, to the previous assay, K1F-GFP and T4 were chosen as two phage representatives of varied morphologies. Host cultures for K1F-GFP (*E. coli* EV36) and T4 (*E. coli* K-12) were grown from a starting concentration of 1×10^6 CFU.mL^{-1} for 4 hours at 37°C to reach log phase. For the non-polymer phage controls, a further 20 µL of SM-II buffer was added to account for the difference in media volume of corresponding wells. In addition, 1% v/v Chemgene was added as an internal positive control. All samples were grown in a total of 200 µL media. The data was obtained and blank corrected using the MARS analysis software (Version 5.10).

E. coli **poly(sodium 4-styrenesulfonate) growth curves.**

As an additional control assay, another antiviral polymer, poly(sodium 4-styrenesulfonate) was adopted to investigate its antiviral properties against our library of bacteriophages. In short, the standardised growth curve assay was followed, with 24 hour incubation of the bacteriophages K1F-GFP, K1E, K1-5, T7 and T4 in 5 mg.mL^{-1} and 10 mg.mL^{-1} polystyrene sulfonate. The data was obtained and blank corrected using the MARS analysis software as a biological single replicate.

K1F-GFP bacteriophage aggregation assay.

Dynamic light scattering (DLS) measurements were carried out for poly(acrylic acid) incubated K1F-GFP phage to investigate any potential aggregation due to the polymer. Phage and polymer aliquot (1 mL) was prepared, with K1F-GFP phage final concentration of 1.28 × 10^{10} PFU.mL^{-1}, and final poly(acrylic acid) PAA 153 concentration of 10 mg.mL^{-1}, which was incubated for 4 hours. Additional PAA and K1F-GFP controls in SM-II buffer were also prepared, with matching concentrations to the original condition, and incubated for the same duration before testing. Measurements were carried out in technical triplicate on a Zetasizer ZS instrument and analysed using Malvern DTS 7.03 software.

Supplementary characterization data for PPEGMA, PMA, PAA, PHEA and PNIPAM homopolymers.

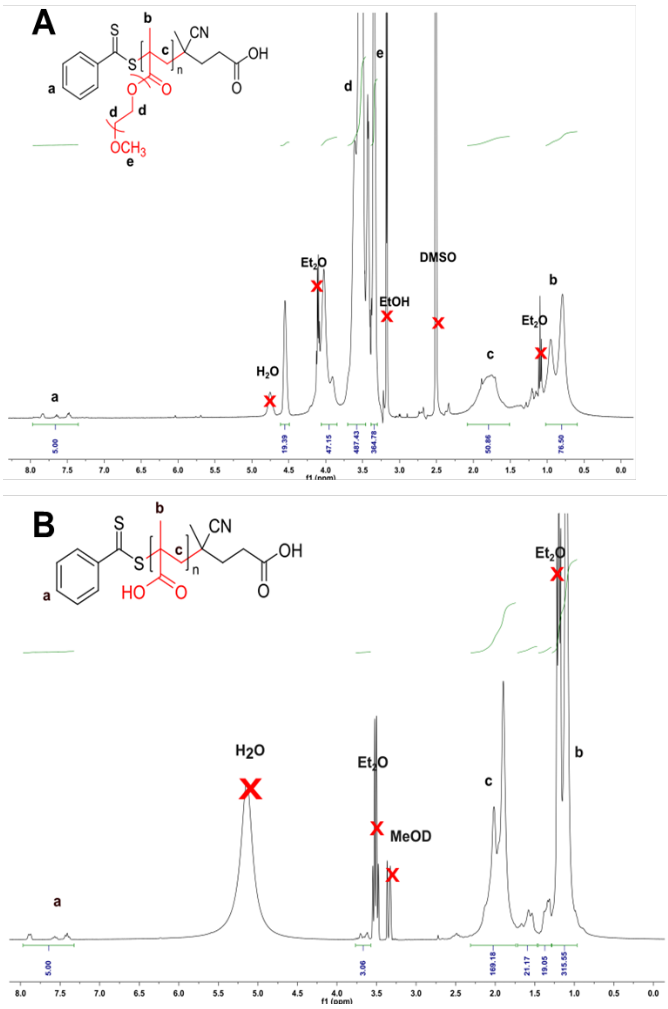

Figure S2. Representative polymer ^{1}H-NMR spectra. A) ^{1}H-NMR spectrum of Poly(poly(ethylene glycol) methacrylate) $PPEGMA_{100}$ (coded PPEGMA 77) (400 MHz, DMSO-d_6); B) ^{1}H-NMR spectrum of poly(methacrylic acid) PMA_{100} (coded PMA 213) (400 MHz, MeOD-d_4).

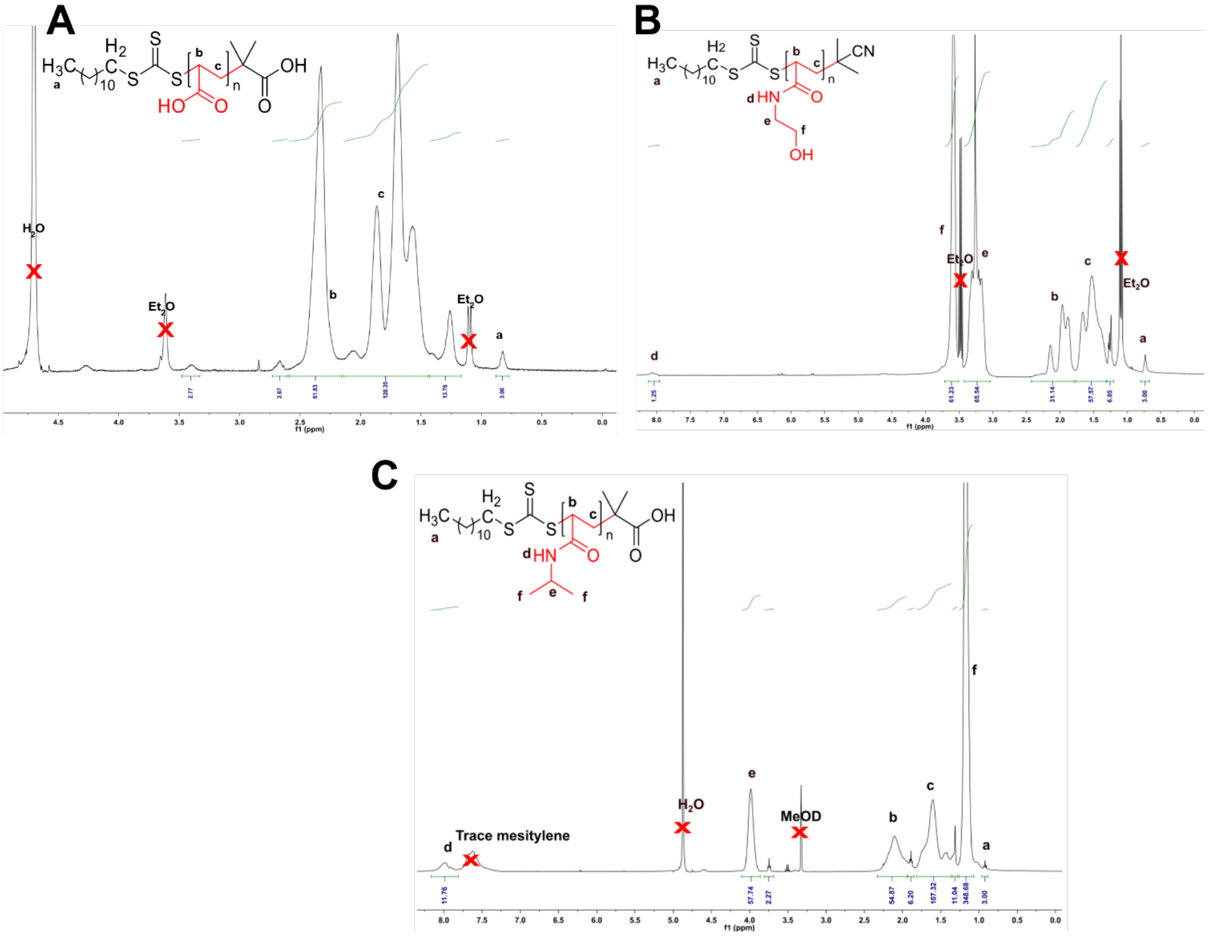

Figure S3. Representative polymer ^{1}H-NMR spectra. A) ^{1}H-NMR spectrum of poly(acrylic acid) PAA_{100} (coded PAA 153) (400 MHz, MeOD-d_4); B) ^{1}H-NMR spectrum of poly(*N*-hydroxyethyl acrylamide) $PHEA_{100}$ (coded PHEA 78) (400 MHz, MeOD-d_4); C) ^{1}H-NMR spectrum of poly(*N*-isopropyl acrylamide) $PNIPAM_{100}$ (coded PNIPAM 96) (400 MHz, MeOD-d_4).

High throughput testing of the polymer library with the bacteriophages.

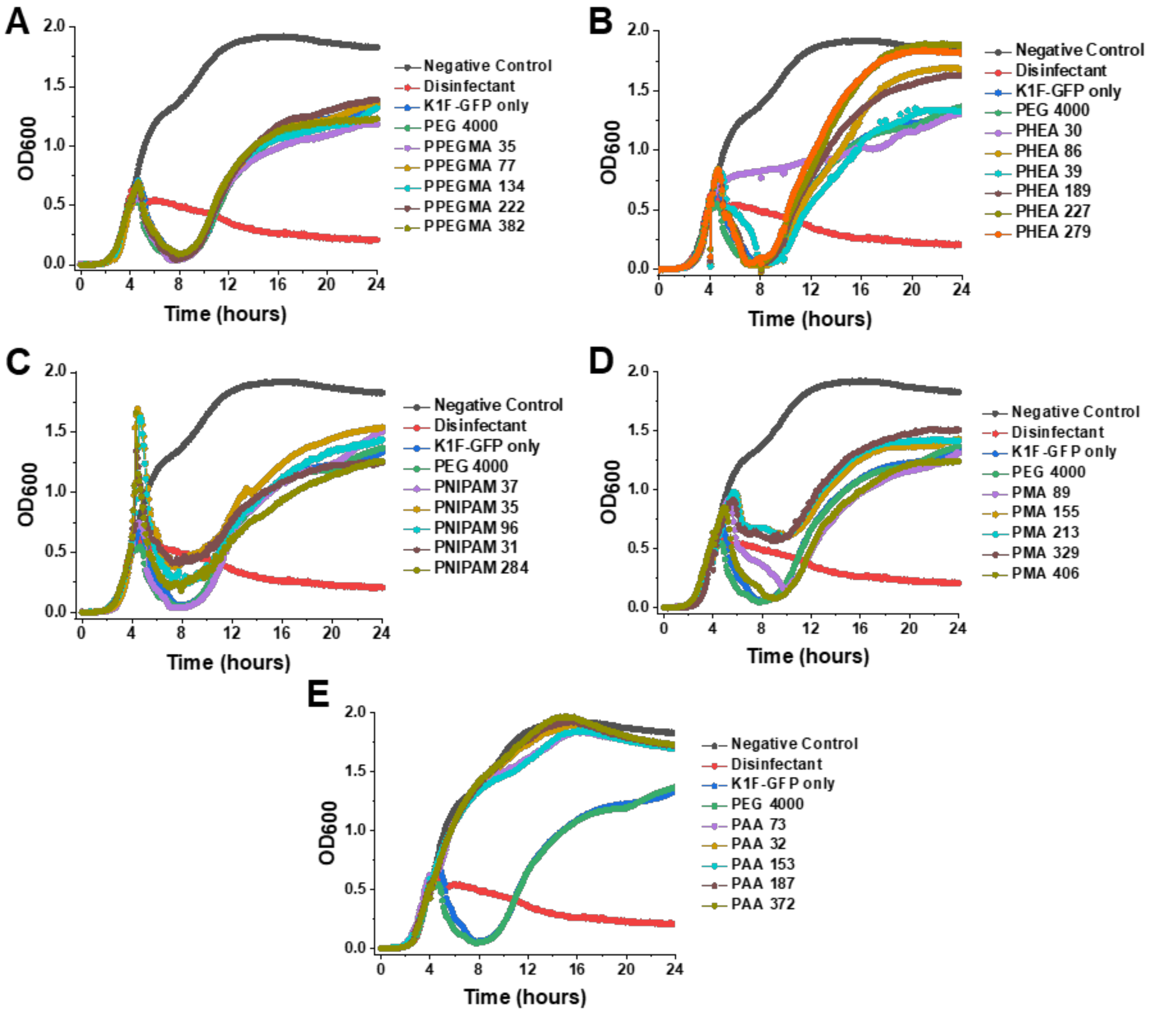

Figure S4. Screening for K1F-GFP bacteriophage inhibition. Growth curves with inactive polymers: A) PPEGMA B) PHEA, C) PNIPAM, D) PMA. E) Growth curve with PAA, the only 'hit' from the screen. *E. coli* EV36 was used as the bacteria host, with a starting concentration of 1×10^6 CFU.mL^{-1}, and phages were added during the log phase (4 h). PEG 4000 and polymer codes refer to overnight incubated phages in the respective polymer at 10 mg.mL^{-1}, K1F-GFP only refers to SM-II buffer incubated bacteriophage. 1% Chemgene laboratory disinfectant was used as positive and LB media as negative control. The growth curves represent three biological and two technical replicates.

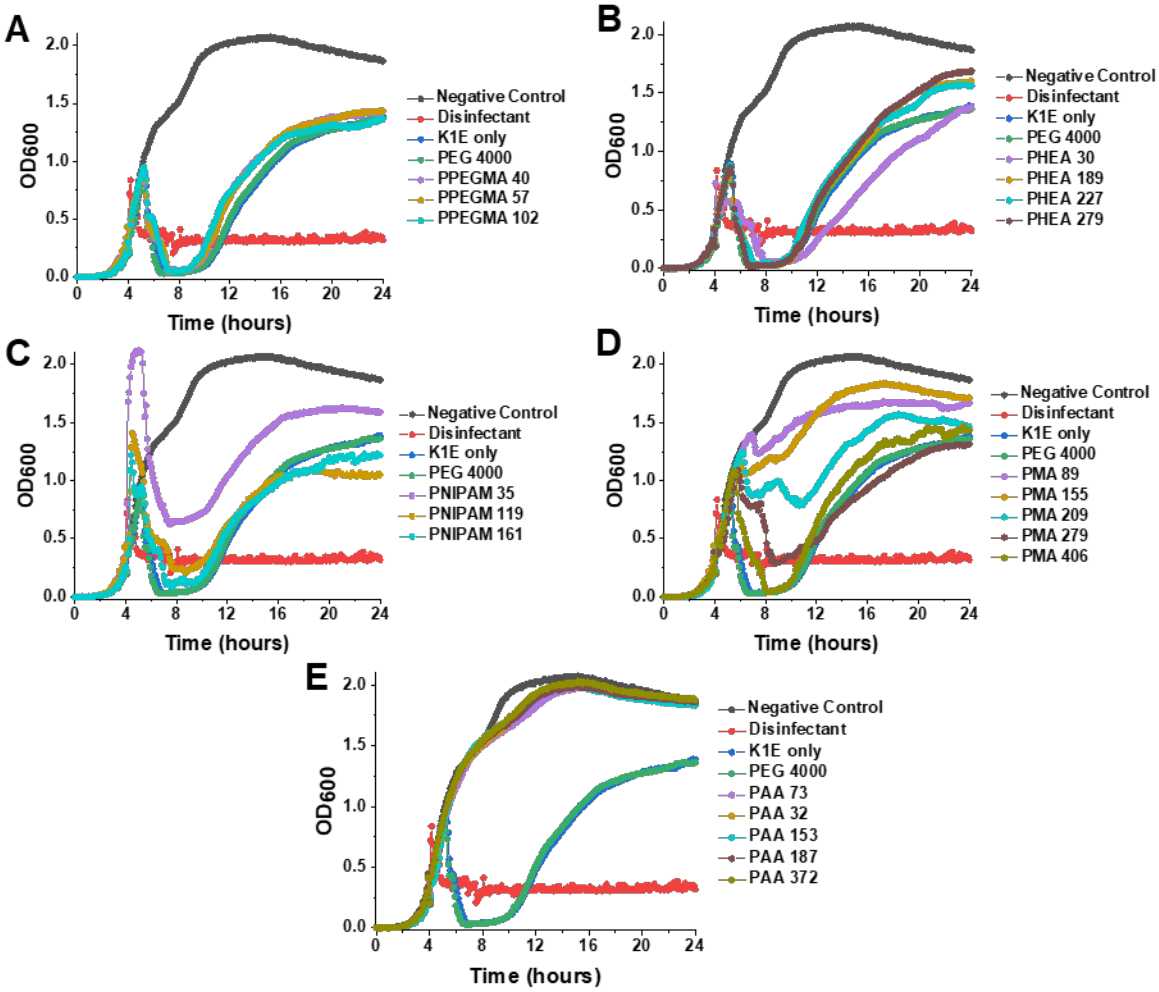

Figure S5. Polymer screening for K1E bacteriophage inhibition. Growth curves with inactive polymers: A) PPEGMA B) PHEA, C) PNIPAM, D) PMA. E) Growth curve with poly(acrylic acid), the only ‘hit’ from the screening. Similarly, to K1F-GFP, *E. coli* EV36 was used as the bacteria host, with a starting concentration of 1×10^6 CFU.mL^{-1}, and phages were added during the log phase. PEG 4000 and polymer codes refer to overnight incubated phages in the respective polymer (10 mg.mL^{-1}), K1E only refers to SM-II buffer incubated bacteriophage. 10% Chemgene laboratory disinfectant was used as positive and LB media as negative control. Each growth curve represents one biological and three technical replicates.

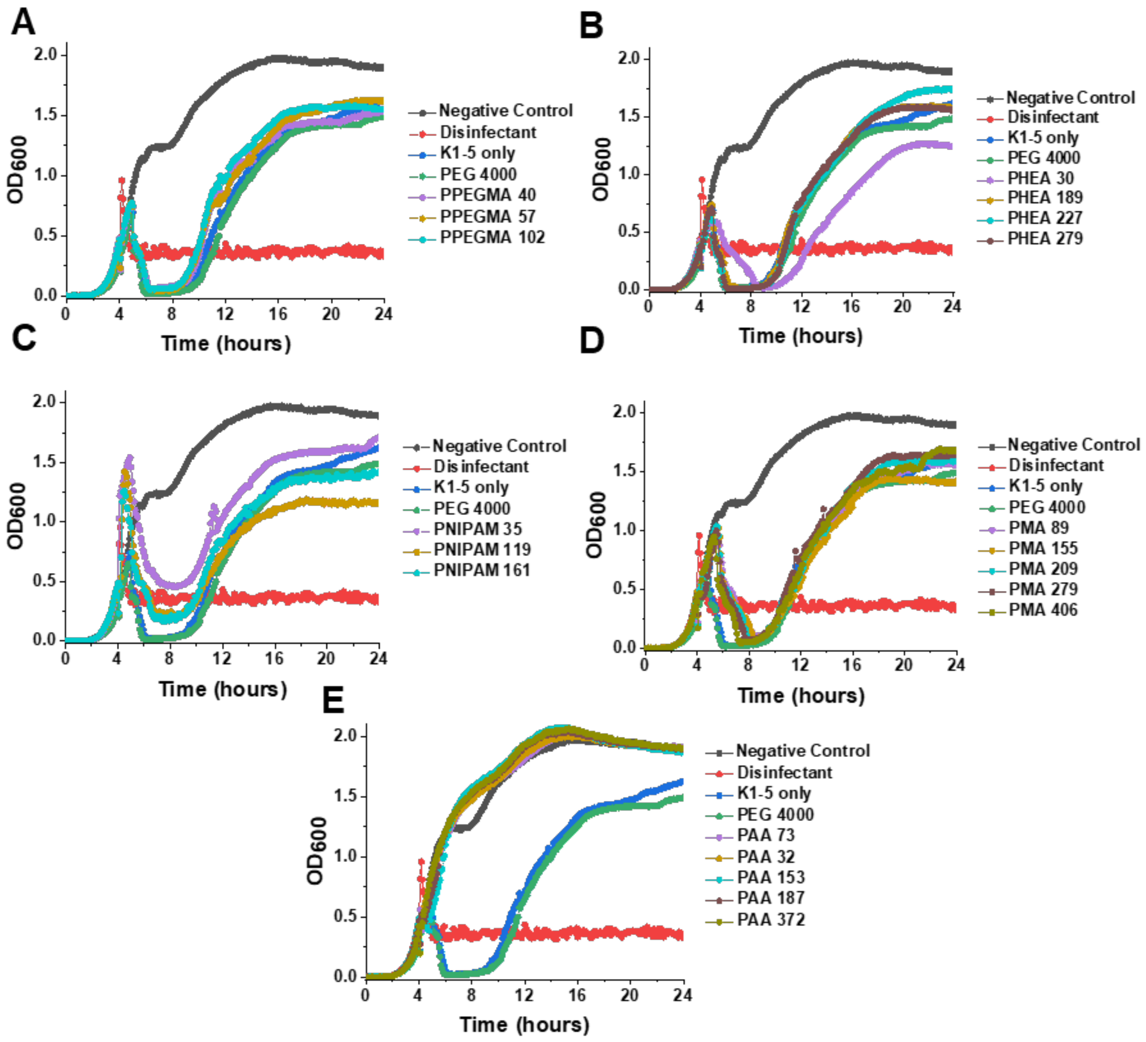

Figure S6. Screening for K1-5 bacteriophage inhibition. Growth curves of the inactive polymers: A) PPEGMA B) PHEA, C) PNIPAM, D) PMA. E) Growth curve of K15 with PAA, the only 'hit' from the screening. In all three 'K' phages, *E. coli* EV36 was used as the bacteria host, with a starting concentration of 1 × 10^6 CFU.mL^{-1}, and phages were added during the log phase (4 h). PEG 4000 and polymer codes refer to overnight incubated phages in the respective polymer with a concentration of 10 mg.mL^{-1}, whereas K1-5 only refers to SM-II buffer incubated bacteriophage. 10% Chemgene laboratory disinfectant was used as positive and LB media as negative control. The growth curves represent one biological and three technical replicates.

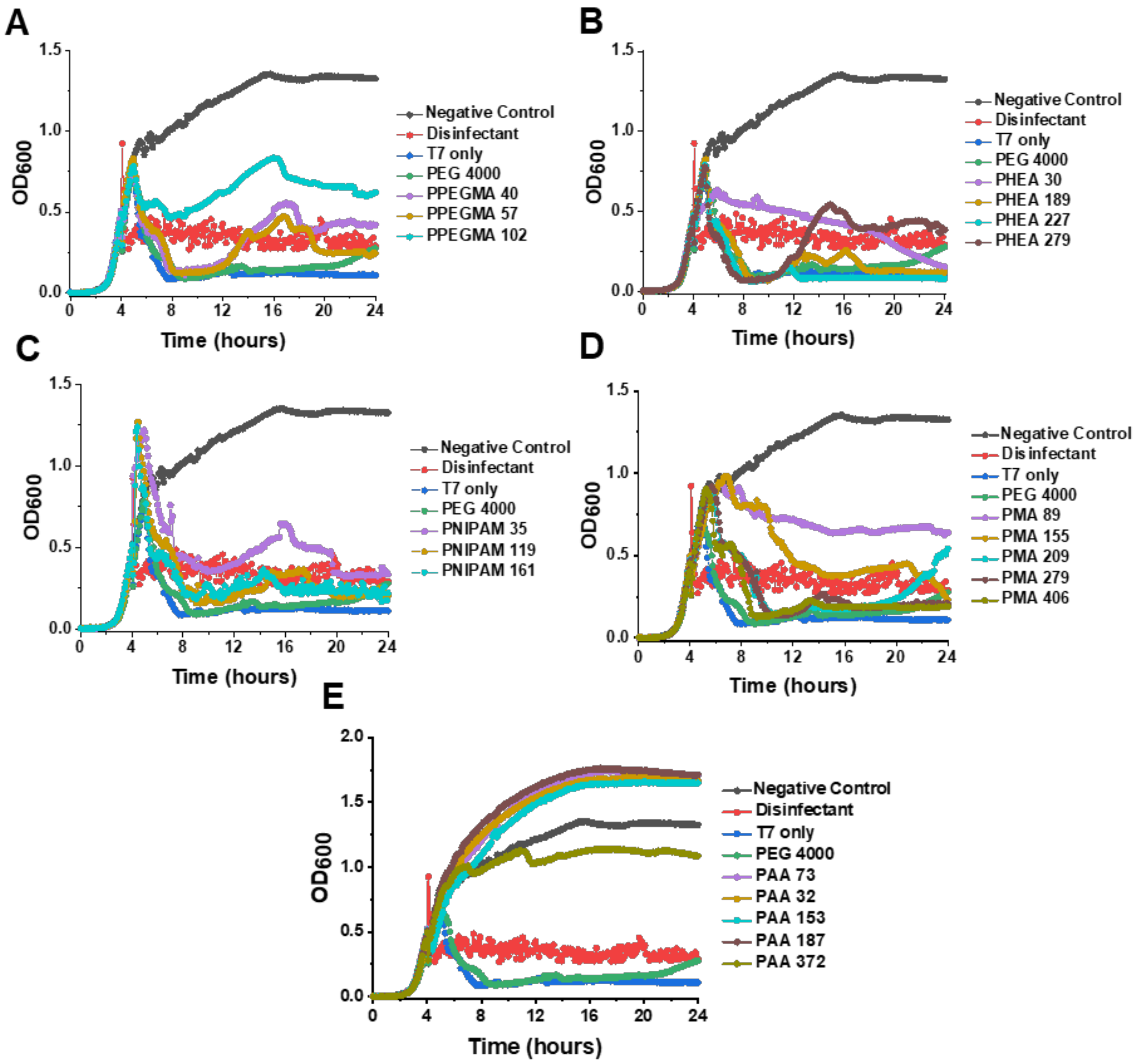

Figure S7. Polymer screening for T7 bacteriophage inhibition. Growth curves with the inactive polymers: A) PPEGMA B) PHEA, C) PNIPAM, D) PMA. E) Growth curve with poly(acrylic acid), the only 'hit' from the screen. *E. coli* K-12 (MG1655 cells) was used as the bacteria host, with a starting concentration of 1×10^6 CFU.mL^{-1}, and phages were added during the log phase. PEG 4000 and polymer codes refer to overnight incubated phages in the respective polymer at a concentration of 10 mg.mL^{-1}, T7 only refers to SM-II buffer incubated bacteriophage. 10% Chemgene disinfectant was used as positive and LB media only as negative control. Each growth curve represents one biological and three technical replicates.

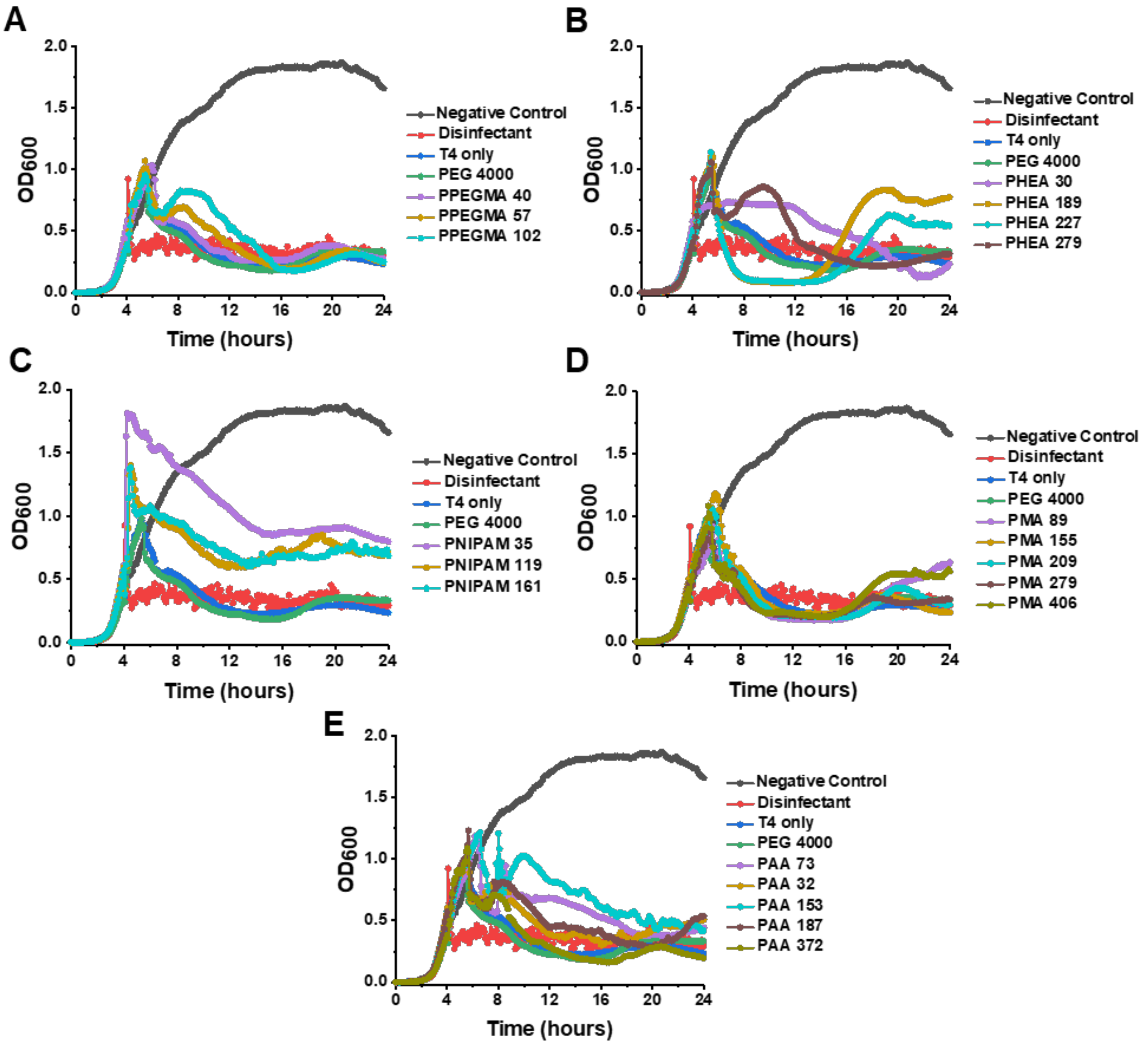

Figure S8. Screening for T4 bacteriophage inhibition. Growth curves of the inactive polymers: A) PPEGMA B) PHEA, C) PNIPAM, D) PMA. E) Growth curve with PAA, the only 'hit' from the screening. Similarly, to T7, *E. coli* K-12 (MG1655 cells) was used as the bacteria host, with a starting concentration of 1×10^6 CFU.mL^{-1}, and phages were added during the log phase of 4 hours. PEG 4000 and polymer codes refer to overnight incubated phages in the respective polymer (10 mg.mL^{-1}), T4 only refers to SM-II buffer incubated bacteriophage. 10% Chemgene disinfectant was used as positive and LB media as negative control. The growth curves represent one biological and three technical replicates.

Poly(acrylic acid) dose response growth curves.

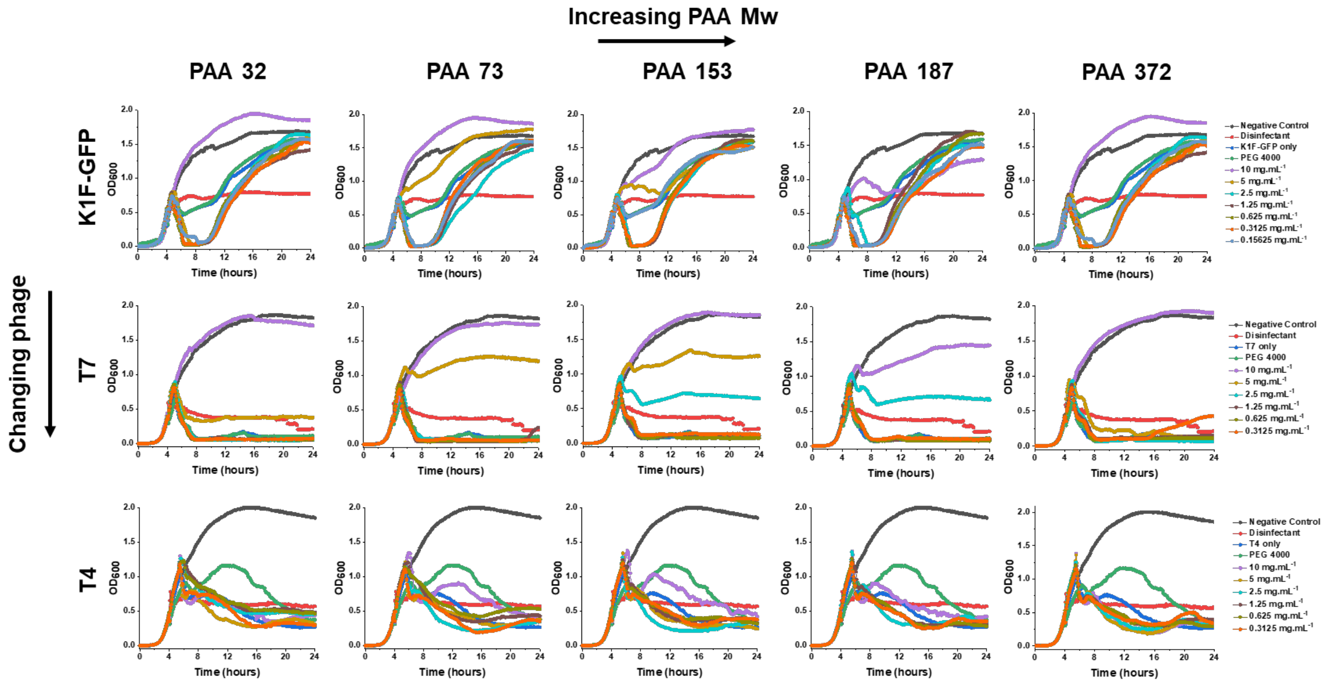

Figure S9. Poly(acrylic acid) dose response bacteriophage inhibition. Dose response growth curves of bacteriophages K1F-GFP, T7 and T4 (from top to bottom) incubated in PAA 32, PAA 73, PAA 153, PAA 187 and PAA 372 (from left to right). The dilution series ranged from 10 mg.mL^{-1} to 0.15625 mg.mL^{-1} PAA for K1F-GFP phage and 10 mg.mL^{-1} to 0.3125 mg.mL^{-1} PAA for T7 and T4 phages. *E. coli* EV36 was used as the bacteria host for K1F-GFP phage, whereas *E. coli* K-12 (MG1655 cells) was used as bacteria host for both T7 and T4 phages, with a starting concentrations of 1 × 10^{6} CFU.mL^{-1}, and phages were added during the log phase (4 h). The various molecular weights of poly(acrylic acid) were serially diluted from before adding the phages to incubate overnight. Additionally, phages were incubated in 10 mg.mL^{-1} poly(ethylene glycol) Mn 4000, or simply SM-II overnight. 1% Chemgene disinfectant was used as positive and LB media as negative control. The dose response growth curves represent three biological and two technical replicates.

Poly(methacrylic acid) incubated bacteriophage plaque assays.

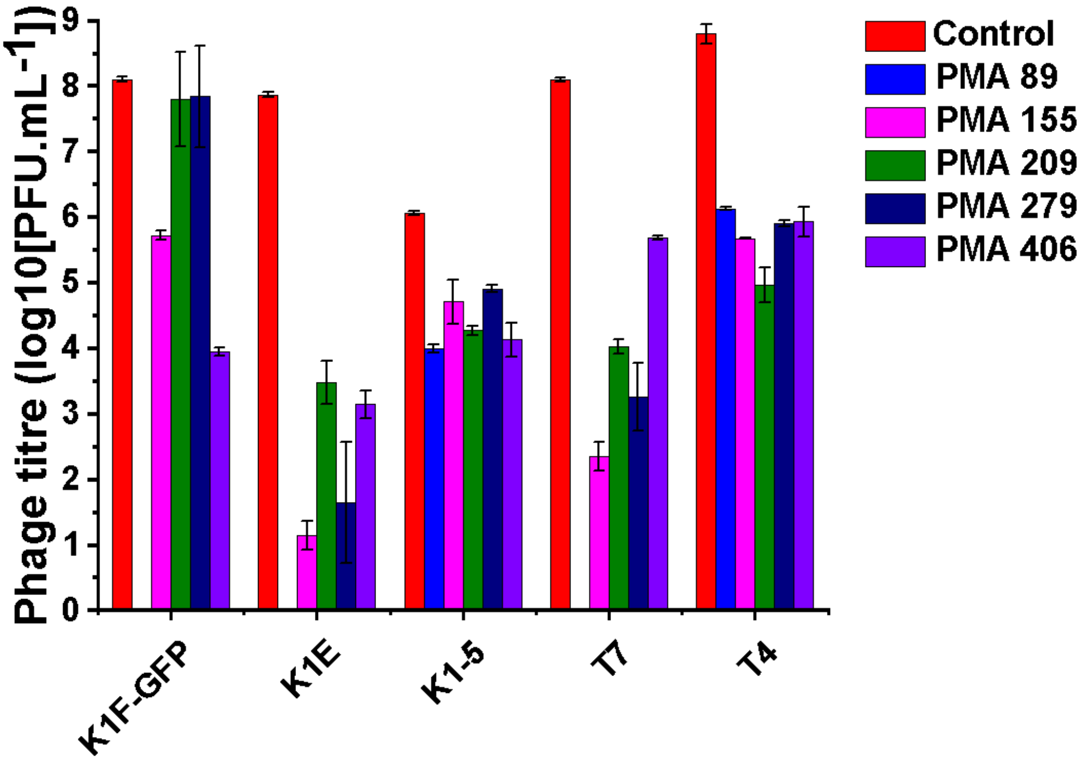

Figure S10. Poly(methacrylic acid) 96-hour incubated bacteriophage plaque assay. *E. coli* EV36 was used as host for K1F-GFP, K1E and K1-5 phages; *E. coli* K-12 was used as host for T7 and T4 phages. Phages were incubated in 10 mg.mL^{-1} of poly(methacrylic acid) (PMA) or SM-II buffer only as a negative control. No plaques observed for K1G-GFP, K1E and T7 when incubated with PMA 89. Assay represents one biological replicate and technical duplicates.

Electron microscopy of bacteriophages K1F-GFP and T4.

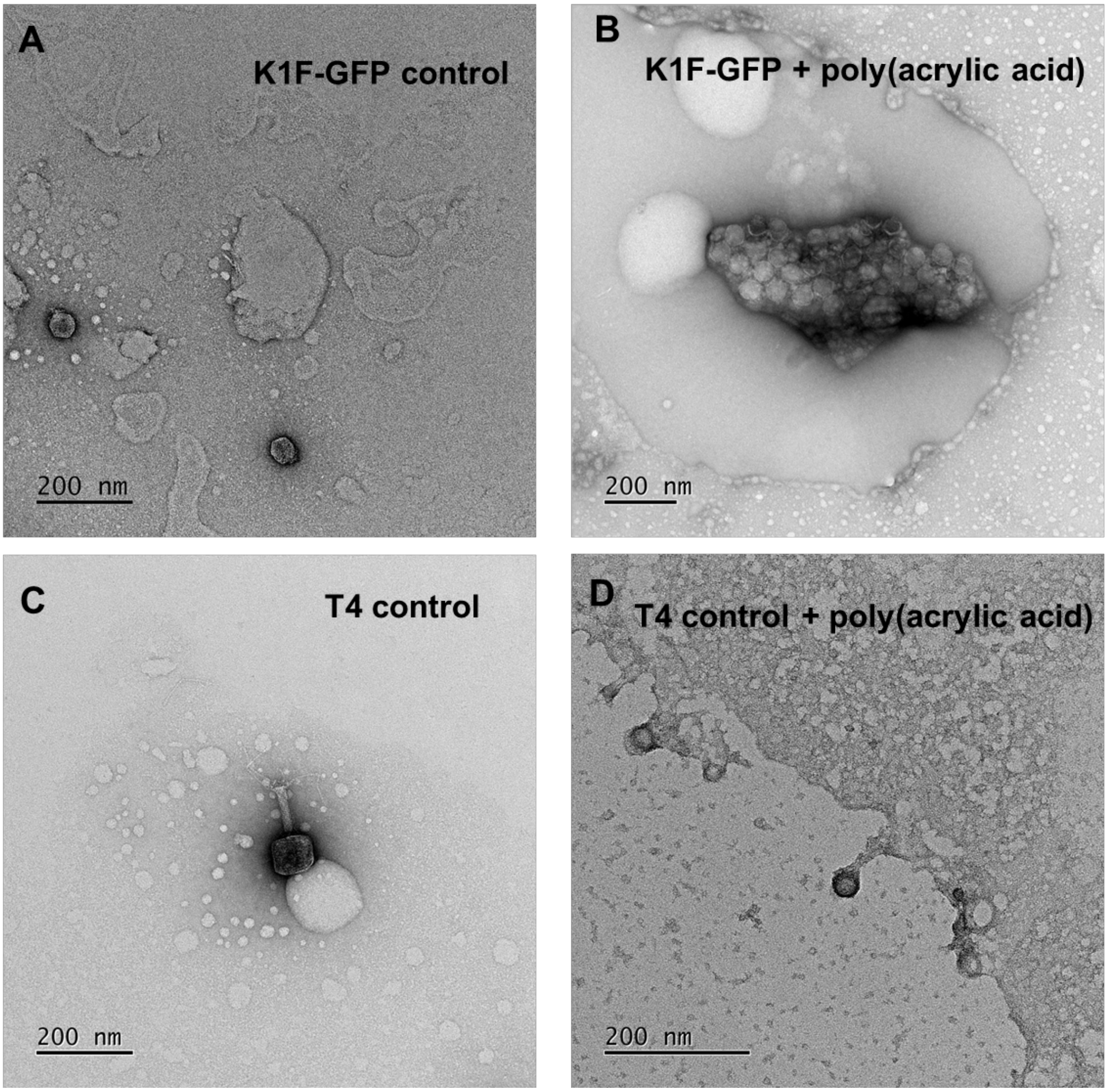

Figure S11. Negative staining Electron micrographs of K1F-GFP and T4 bacteriophages. A) K1F-GFP phage control; B) K1F-GFP phage incubated in 10 mg.mL^{-1} poly(acrylic acid) PAA 153 for 1 hour; C) T4 phage control; D) T4 phage incubated in PAA 153 (10 mg.mL^{-1}) for 1 hour. K1F-GFP and T4 controls were obtained as 40× magnification. PAA incubated K1F-GFP image was obtained as 30× whereas T4 image at 60× magnification. Scale bars are 200 nm.

***Escherichia coli* recombinant green fluorescent protein (GFP) expression.**

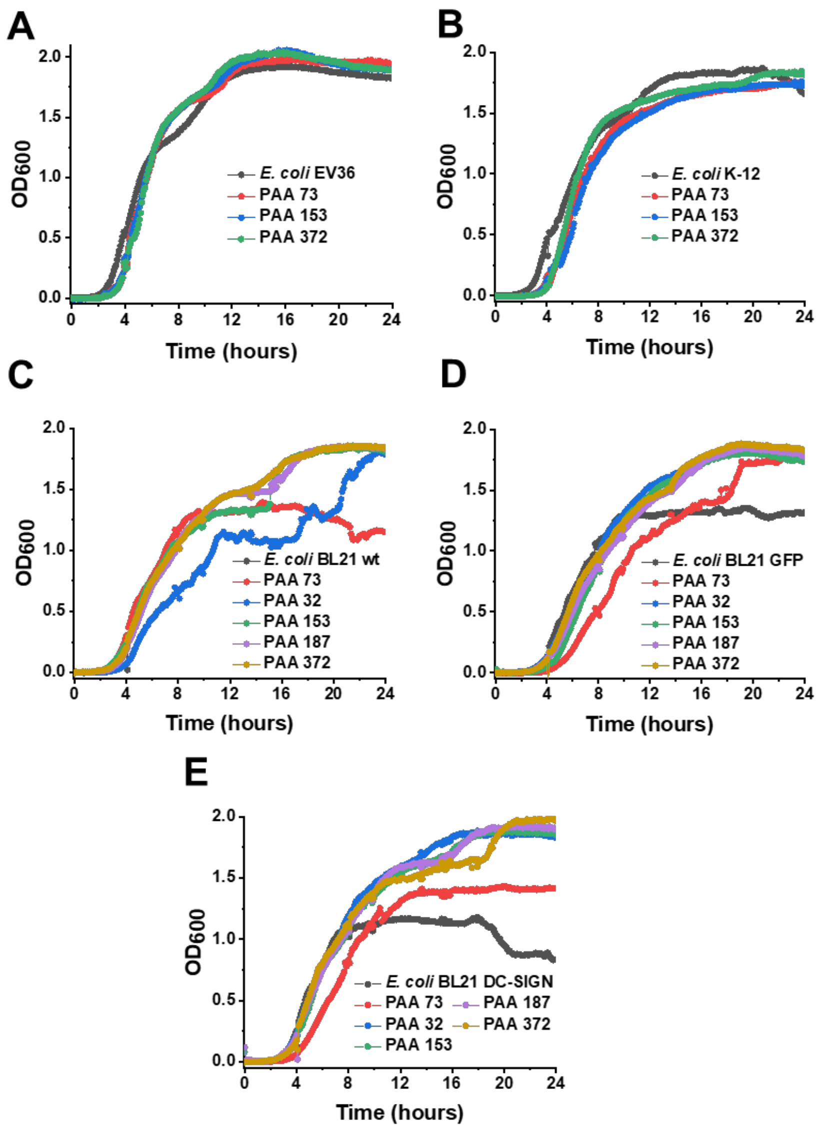

Figure S12. Poly(acrylic acid) screening for bacterial toxicity. *E. coli* growth curves for strains: A) EV36 wild type; B) K-12 (MG1655 cells); C) BL21 wild type (DE3 cells); D) BL21 containing pWALDO plasmid encoding for hexahistidine-tagged green fluorescent protein (GFP); E) BL21 containing pT5T plasmid encoding for the human lectin DC-SIGN. *E. coli* strains had a starting concentration of 1×10^6 CFU.mL^{-1}. Black lines represent the non-polymer containing negative controls. Each growth curve represents three biological and three technical replicates.

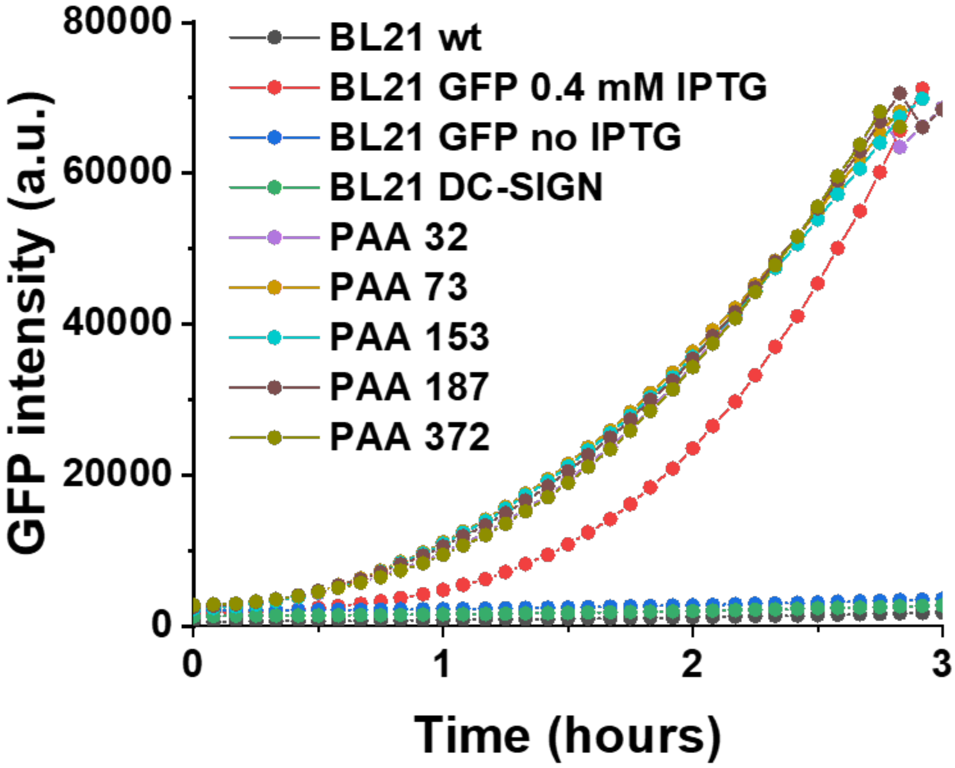

Figure S13. *E. coli* green fluorescent protein (GFP) expression growth curves. Poly(acrylic acid) (PAA) (10 mg.mL^{-1}) of varied molecular weights was added to *E. coli* BL21 containing the GFP expressing pWALDO plasmid (BL21 GFP), at final concentration of 2×10^8 CFU.mL^{-1}, followed by the addition of IPTG inducer. *E. coli* BL21 wild type and BL21 containing the pT5T plasmid encoding for human lectin DC-SIGN strains (non-GFP) and the pWALDO plasmid containing strain without the inducer were used as controls for this assay. Each curve represents three biological and three technical replicates.

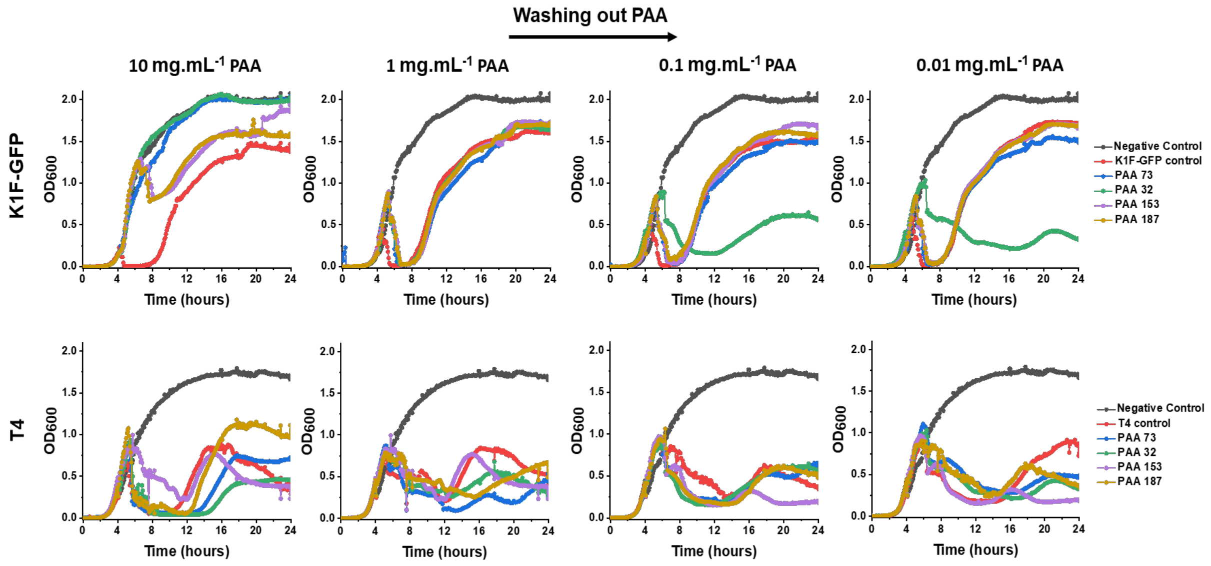

Figure S14. Virustatic verses virucidal experiment for PAA. Post-wash (from left to right) dose response growth curves of bacteriophages K1F-GFP (top row) and T4 (bottom row). The phages were first incubated in 10 mg.mL^{-1} PAA 73, PAA 32, PAA 153 and PAA 187 for 24-hours before polymer washing out by dilution to the indicated concentration and addition of aliquot to log phage (4 h) *E. coli* cultures. *E. coli* EV36 was used as the bacteria host for K1F-GFP phage, whereas *E. coli* K-12 (MG1655 cells) was used as bacteria host for T4 phages, with starting concentration of 1×10^6 CFU.mL^{-1} . K1F-GFP and T4 controls refer to diluted non-polymer containing bacteriophage aliquots which matched the PFU.mL^{-1} (plaque forming units) of each PAA sample within the same condition, whereas LB media was used as negative control. Phage inhibition is only seen around the MIC, but no inhibition once the polymer has been diluted. The dose response growth curves represent one biological and two technical replicates.

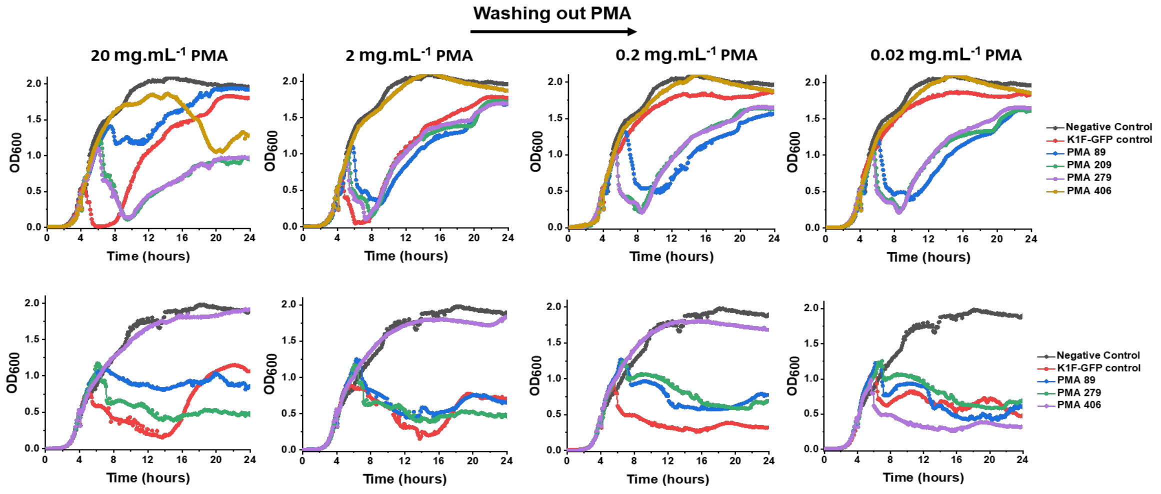

Figure S15. Virustatic verses virucidal experiment for PMA. Post-wash (left to right) dose response growth curves of bacteriophages K1F-GFP (top row) and T4 (bottom row). The phages were incubated in 20 mg.mL^{-1} PMA 89, PMA 209 (only K1F-GFP), PMA 279 and PMA 406 for 72-hours before polymer washing out by 1:10 dilution and addition of the aliquot to log phage (4 h) host cultures. *E. coli* EV36 was used as the bacteria host for K1F-GFP phage, whereas *E. coli* K-12 (MG1655 cells) was used as bacteria host for T4 phages, with starting concentration of 1×10^6 CFU.mL^{-1} . K1F-GFP and T4 controls refer to the diluted non-polymer containing bacteriophage aliquots which matched the PFU.mL^{-1} (plaque forming units) of each PMA sample within the same condition, whereas LB media was used as negative control. The dose response growth curves represent one biological and two technical replicates.

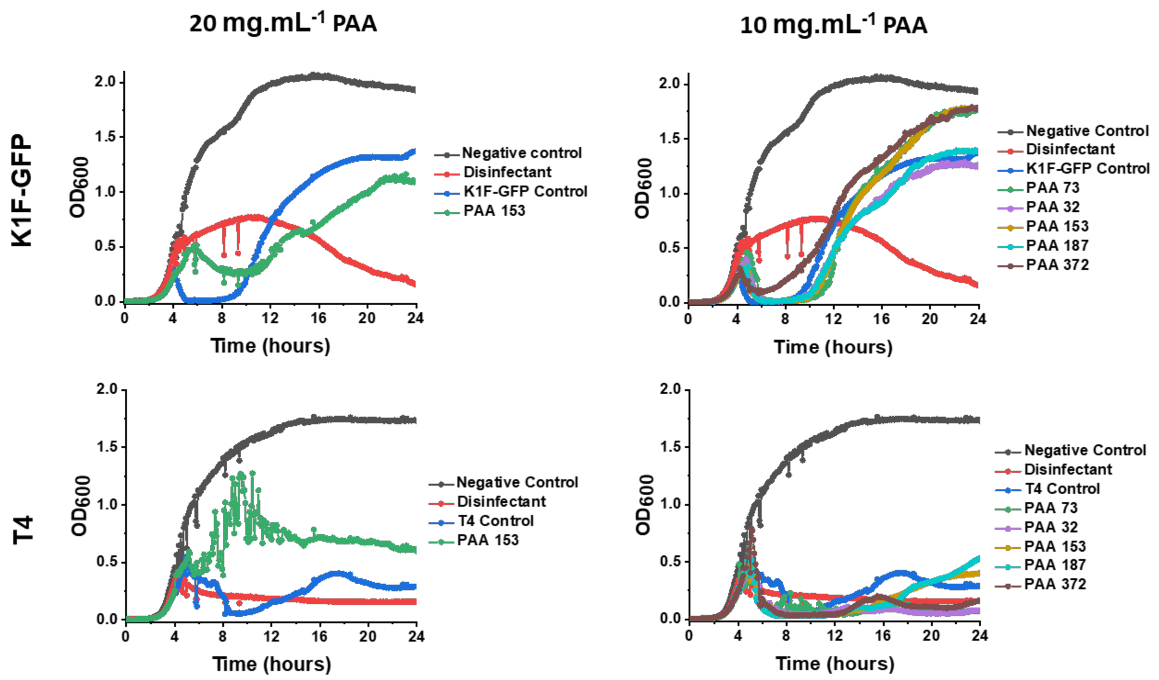

Figure S16. Poly(acrylic acid) post infection ('therapeutic') phage inhibition experiment. Post-infection polymer growth curves of bacteriophages K1F-GFP (top row) and T4 (bottom row), with 20 mg.mL^{-1} PAA (LHS column) and 10 mg.mL^{-1} PAA (RHS column). The phages were first added to the log phage (4 h) host cultures, followed by the addition of the poly(acrylic acid) aliquot. *E. coli* EV36 was used as the bacteria host for K1F-GFP phage, whereas *E. coli* K-12 (MG1655 cells) was used as bacteria host for T4 phages, with starting concentration of 1 × 10^{6} CFU.mL^{-1} . K1F-GFP and T4 controls refer to non-polymer containing bacteriophage aliquots, whereas LB media was used as negative control. Positive control used was 1% v/v Chemgene. The growth curves represent one biological and two technical replicates.

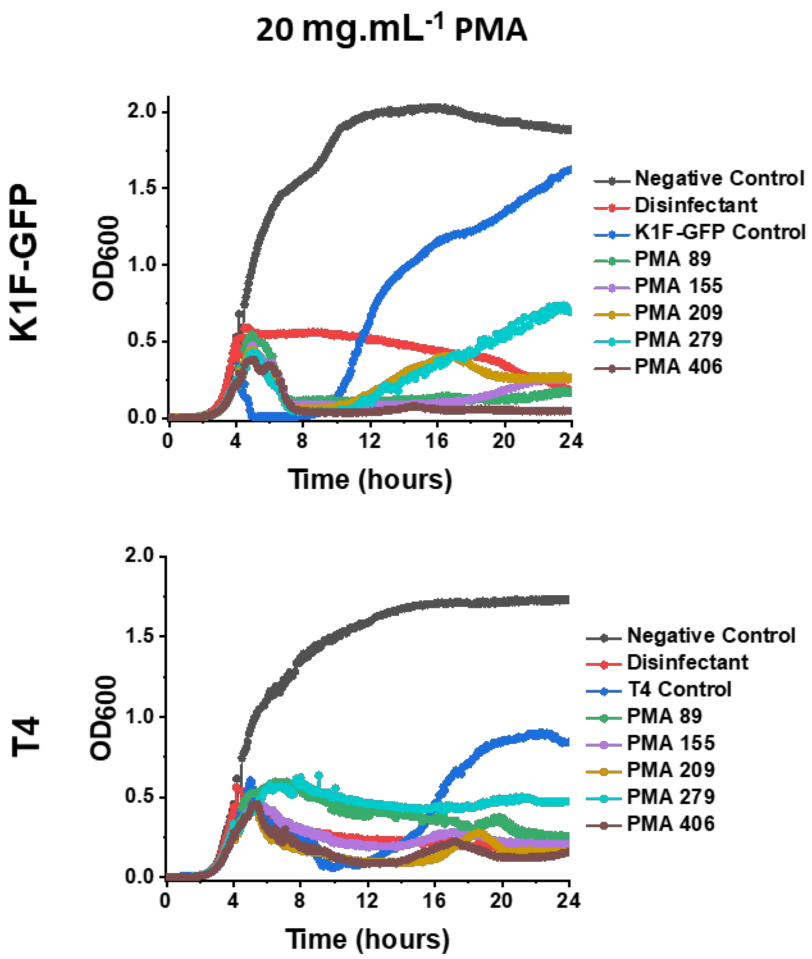

Figure S17. Poly(methacrylic acid) post infection ('therapeutic') phage inhibition. Post-infection polymer growth curves of bacteriophages K1F-GFP (top) and T4 (bottom), with 20 mg.mL^{-1} PMA. The phages were first added to the log phage (4 h) host cultures, followed by the addition of the poly(methacrylic acid) aliquot. *E. coli* EV36 was used as the bacteria host for K1F-GFP phage, whereas *E. coli* K-12 (MG1655 cells) was used as host for T4 phages, with starting concentration of 1×10^6 CFU.mL^{-1} . K1F-GFP and T4 controls refer to non-polymer containing phage aliquots, whereas LB media was used as negative control. 1% v/v Chemgene was used as disinfectant (positive control). The growth curves represent one biological and two technical replicates.

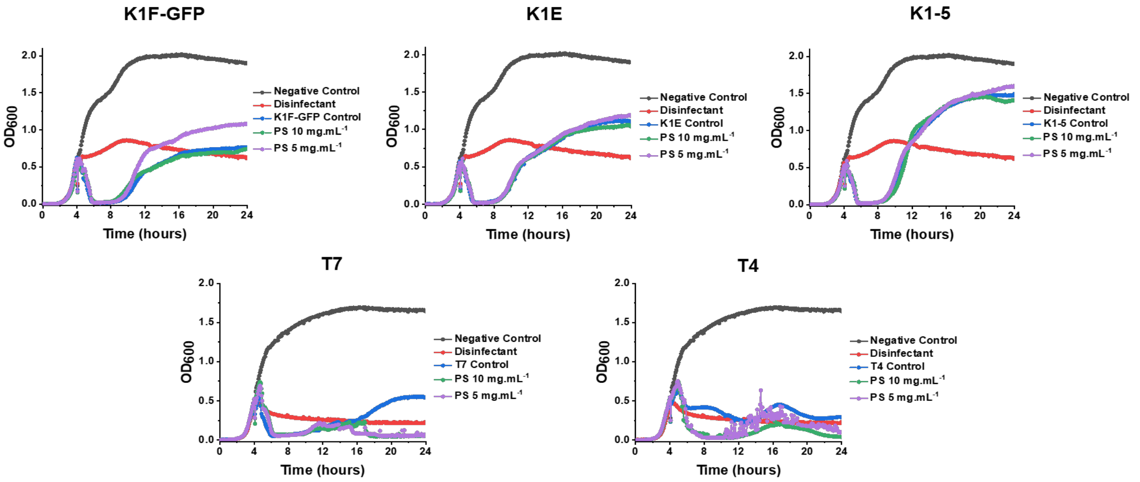

Figure S18. Poly(sodium 4-styrenesulfonate) bacteriophage inhibition. Growth curves of 24 hour incubated K1F-GFP, K1E, K1-5, T7 and T4 bacteriophages (top left to bottom right) in 5 mg.mL^{-1} and 10 mg.mL^{-1} poly(sodium 4-styrenesulfonate) SM-II buffer. Post-incubation phage aliquots were added to log phage host cultures. *E. coli* EV36 was used as the bacteria host for K1F-GFP, K1E and K1-5 phages, whereas *E. coli* K-12 (MG1655 cells) was used as host for T7 and T4 phages, with starting concentration of 1 × 10^{6} CFU.mL^{-1} each. Phage controls refer to non-polymer containing bacteriophage aliquots. PS was used as shorthand for polystyrene sulfonate. LB media was used as negative, whereas 1% v/v Chemgene in LB media as disinfectant (positive control). The dose response growth curves represent one biological and two technical replicates.

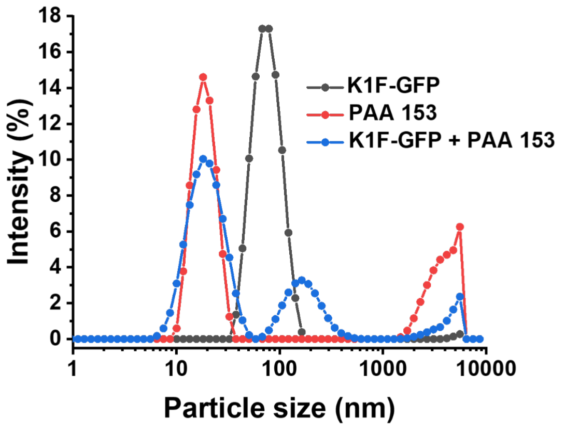

Figure S19. Particle size distributions of K1F-GFP bacteriophage and poly(acrylic acid). K1F-GFP phage final concentration was 1.28×10^{10} PFU.mL^{-1} in both before and after polymer conditions. Poly(acrylic acid) (PAA 153) final concentration was 10 mg.mL^{-1} in both conditions. The K1F-GFP phage aliquot was incubated in PAA 153 for 4 hours before analysis. The measurements represent one biological and three technical replicates.

References

(1) Richards, S.-J.; Gibson, M. I. Optimization of the Polymer Coating for Glycosylated Gold Nanoparticle Biosensors to Ensure Stability and Rapid Optical Readouts. *ACS Macro Lett.* **2014**, *3* (10), 1004–1008.

(2) Pancaro, A.; Szymonik, M.; Georgiou, P. G.; Baker, A. N.; Walker, M.; Adriaensens, P.; Hendrix, J.; Gibson, M. I.; Nelissen, I. The Polymeric Glyco-Linker Controls the Signal Outputs for Plasmonic Gold Nanorod Biosensors Due to Biocorona Formation. *Nanoscale* **2021**, *13* (24), 10837–10848.

(3) Cooper, P. D. The Plaque Assay of Animal Viruses; 1962; pp 319–378.

(4) Cagno, V.; Andreozzi, P.; D'Alicarnasso, M.; Silva, P. J.; Mueller, M.; Galloux, M.; Goffic, R. Le; Jones, S. T.; Vallino, M.; Hodek, J.; Weber, J.; Sen, S.; Janecek, E. R.; Bekdemir, A.; Sanavio, B.; Martinelli, C.; Donalisio, M.; Welti, M. A. R.; Eleouet, J. F.; Han, Y.; Kaiser, L.; Vukovic, L.; Tapparel, C.; Král, P.; Krol, S.; Lembo, D.; Stellacci, F. Broad-Spectrum Non-Toxic Antiviral Nanoparticles with a Virucidal Inhibition Mechanism. *Nat. Mater.* **2018**, *17* (2), 195–203.

(5) Kocabiyik, O.; Cagno, V.; Silva, P. J.; Zhu, Y.; Sedano, L.; Bhide, Y.; Mettier, J.; Medaglia, C.; Da Costa, B.; Constant, S.; Huang, S.; Kaiser, L.; Hinrichs, W. L. J.; Huckriede, A.; Le Goffic, R.; Tapparel, C.; Stellacci, F. Non-Toxic Virucidal Macromolecules Show High Efficacy Against Influenza Virus Ex Vivo and In Vivo. *Adv. Sci.* **2021**, *8* (3), 1–8.
